# Assessing the communication gap between AI models and healthcare professionals: Explainability, utility and trust in AI-driven clinical decision-making

**DOI:** 10.1016/j.artint.2022.103839

**Published:** 2023-03

**Authors:** Oskar Wysocki, Jessica Katharine Davies, Markel Vigo, Anne Caroline Armstrong, Dónal Landers, Rebecca Lee, André Freitas

**Affiliations:** a Department of Computer Science, The University of Manchester, United Kingdom of Great Britain and Northern Ireland; b Digital Experimental Cancer Medicine Team, Cancer Biomarker Centre, CRUK Manchester Institute, University of Manchester, United Kingdom of Great Britain and Northern Ireland; c Faculty of Biology Medicine and Health, The University of Manchester, United Kingdom of Great Britain and Northern Ireland; d Idiap Research Institute, Switzerland

**Keywords:** Explainable model, Explainable AI, ML in healthcare, User study, Clinical decision support, Automation bias, Confirmation bias, Explanation’s impact

## Abstract

This paper contributes with a pragmatic evaluation framework for explainable Machine Learning (ML) models for clinical decision support. The study revealed a more nuanced role for ML explanation models, when these are pragmatically embedded in the clinical context. Despite the general positive attitude of healthcare professionals (HCPs) towards explanations as a safety and trust mechanism, for a significant set of participants there were negative effects associated with confirmation bias, accentuating model over-reliance and increased effort to interact with the model. Also, contradicting one of its main intended functions, standard explanatory models showed limited ability to support a critical understanding of the limitations of the model. However, we found new significant positive effects which repositions the role of explanations within a clinical context: these include reduction of automation bias, addressing ambiguous clinical cases (cases where HCPs were not certain about their decision) and support of less experienced HCPs in the acquisition of new domain knowledge.

## 1 Introduction

Clinical predictive models based on machine learning (ML) bring the promise of integrating real world evidence into clinical decision-making, balancing individual clinical experience with data-driven evidence. The dialogue between evidence, which is systematically collected and analysed, and in clinical practice, allows for a continuous evolution of the understanding of disease and treatment response. This is particularly important in the context of new diseases (e.g. COVID-19), new treatments or in understanding personalised responses (e.g. multi-morbidity or diverse populations).

Effective communication between healthcare professionals (HCPs) and ML models depends on the ability of the former to have a faithful mental representation of the latter and a critical ability to assess the strengths and limitations of ML models.

*Explainability* [1–6] emerged as a research area aiming to address interpretability bottlenecks in ML models resulting from the fact that many of them can be effective in predicting an outcome, but not in explaining their underlying reasoning, which limits their practical application in critical areas. In recent work by [7] *Verification and Explainability Methods* were identified as one of the three main frontier topics in the area of medical AI (together with *Complex Networks and their Inference* and *Graph Causal models and Counterfactuals*) in order to satisfy the need for trustworthiness at multiple levels of the medical workflow. Whilst recent explainable ML methods [8–15] are instrumenting ML models to be more transparent, the perceived utility and suitability of explanation models has not been systematically investigated in the clinical context.

We aimed to address this research gap by conducting a user study on the effectiveness of current modalities of ML explainability amongst healthcare professionals for data-driven decision support. The study was performed using an explainable ML clinical decision support tool designed to help manage the admissions of patients with cancer and COVID-19: CORONET (COVID-19 Risk in ONcology Evaluation Tool [16]). In contrast to existing explainability studies performed over simplified tasks [17–20], our experiment closely mimicked a real-world clinical scenario [21], where the decision is made based on multiple factors supported by a recommendation.

As part of the contributions, we propose a novel methodology to assess the pragmatic utility of explanations, which accounts for a systematic understanding of the interaction between a clinician’s mental model and the explainable ML model. Additionally, we introduce a framework for evaluating the impact of ML explanations on perceived utility and usability in clinical context [22]. We present the results of an experiment conducted involving 23 HCPs, who were presented with 10 scenarios and had to decide whether to admit/discharge a patient with cancer and COVID-19 supported by a model firstly without an explanation (colour bar + score) and with explanation (+contributing features).

## 2 Methods

### 2.1 Framework for pragmatic evaluation of the model’s explanation

**Fig. 1:**
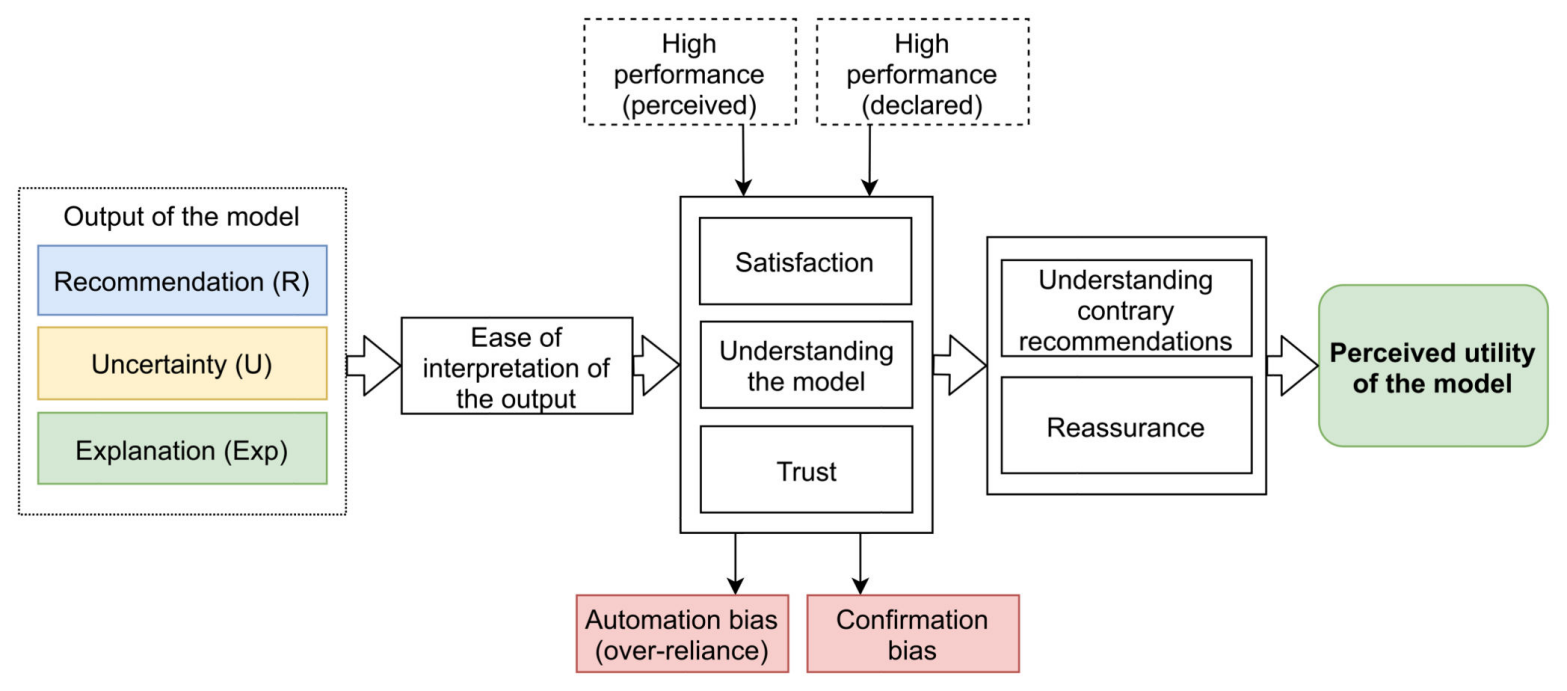
Overview of the framework for pragmatic evaluation of model’s explanation. It highlights key aspects of the user’s mental model that affect the perceived utility of the tool in clinical setting. Pink boxes - negative effects. (For interpretation of the colours in the figures, the reader is referred to the web version of this article.)

We introduce a framework for pragmatic evaluation of the model’s explanation, outlining the key components from the model’s output (including its explanatory components) to the model’s usefulness in a clinical setting (see Fig. 1). It allows for investigating key aspects of the user’s attitude that impact the perceived utility of the model in a clinical decision-making setting. Our end-to-end approach allows for the evaluation of the change in perception of the overall system’s clinical suitability, i.e. evaluation of system-level effect, instead of item-level effect (single recommendation) [21].

In general *explainability* is about transparency and traceability, highlighting decision-relevant parts of the representations within the algorithm, both for the dataset (population) or particular observation (individual patient). *Explanation* is often used interchangeably with *interpretation*, however the distinction must be made as the first is product by the second. *Explanation* is a collection of features from the interpretable domain that contributes to the production of an abstract statement. *Interpretation* is a process of mapping this statement into the domain the human expert can perceive, comprehend, and understand (for more detailed definition refer to [22]). In our framework, first, the ease of interpretation of the tool’s output is evaluated, which is a prerequisite for understanding, satisfaction and trust [23,24]. Note, that in the clinical decision-making process, the cognitive load due to the model output is one of many disjointed contextual factors and thus should not require the full working memory of the user [25].

Then questions regarding trust, satisfaction and model understanding are systematically assessed. Trust in the model leads to its high persuasive power and *reassurance* in cases where the user is less certain about the right decision [26]. Understanding the model’s reasoning process supports the critical assessment of the model (i.e. understanding when the tool provides incorrect recommendations). Satisfaction measures how well the provided output corresponds the user’s expectations.

Trust may be enhanced by providing the explanation [7,27–29], uncertainty of the model’s recommendation [29,26] and/or by ensuring the high performance of the model at a given task [30]. The last aspect can be characterised by the declared performance by the model’s developer in testing and validation, and the subjective performance perceived by the user during the experiment [31,21]. However, excessive trust may have negative effects, i.e. *confirmation bias* [32] and *automation bias* [33,34]. These are evaluated via the concordance between user’s action, recommendation and correct action, and via time spent on decisions.

Finally, all aspects account for the perceived pragmatic utility of the model, evaluated via asking whether HCPs would use the model in their clinical practice. The pragmatic utility aggregates model’s *usability*, defined as how effective, efficient and satisfying the model is in a specified context [22], and *clinical performance*, defined as the ability to deliver results that are correlated to a specific clinical condition and target population in experiments performed by medical experts [35].

The output of the model may consist of three main components: i) recommendation (R, i.e. prediction, prognosis or classification); ii) uncertainty of the model’s recommendation (U); iii) explanation (Exp); and can be presented to the user as: R, R+U, R+Exp and R+U+Exp. In our empirical analysis, as the CORONET model does not communicate its uncertainty, we put aside this aspect and performed a ‘R then R+Exp’ within-subject scenario with one group of HCPs. Although one of the visual components of the explanation (i.e. scatter plot, described in 2.2) makes the user aware of the model’s imperfect performance (predicted scores for the whole training cohort, including incorrect recommendations), we did not evaluate this aspect directly. We did not provide explicitly any performance metric to the HCPs. Similarly, the perceived performance of the model was not measured. Thus, we did not evaluate the contribution of the model’s performance to the HCPs’ attitude towards the model. Individual characteristics of the user, such as user’s background, knowledge and expectations that may impact their attitude [36] were evaluated.

Guided by the framework, we investigate the overall pragmatic impact of the explanations on the clinical decision by targeting the following research questions:

RQ1 Does the explanation improve understanding of the model’s reasoning mechanisms?

RQ2 Does the explanation lead to an increase in model satisfaction?

RQ3 Does the explanation lead to an increase in trust?

RQ4 Do explanations improve the HCPs’ critical understanding of the model?

RQ5 Do explanations enable improved clinical understanding?

RQ6 How do explanations impact the automation bias?

RQ7 Does the explanation increase the confirmation bias?

This study indicates that there is a major unaddressed communication gap between explainable ML models and healthcare professionals and elicits priority areas for investigation.

### 2.2 Clinical setting & supporting model

#### CORONET model

**Fig. 2:**
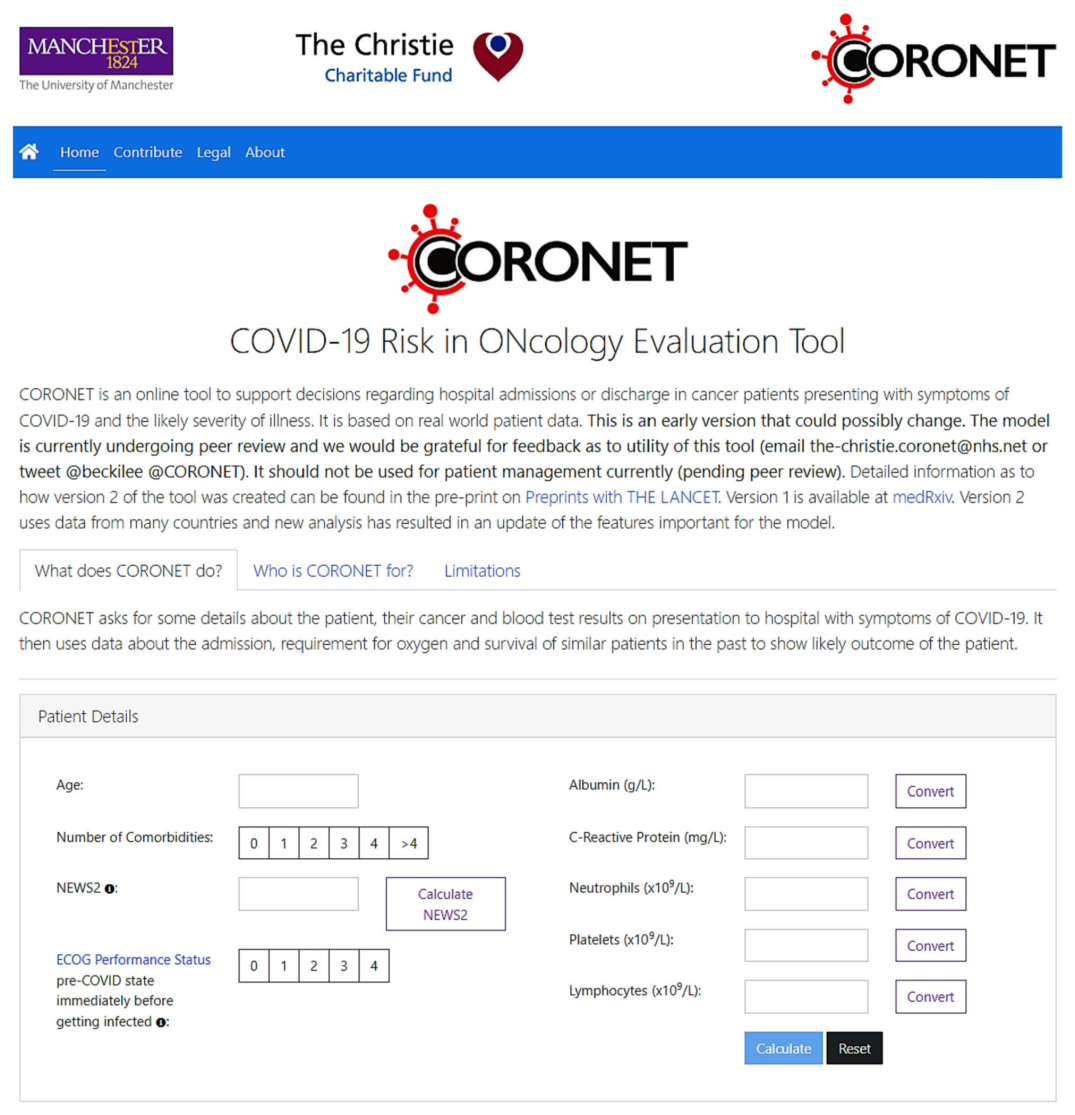
User’s interface of the CORONET available at https://coronet.manchester.ac.uk. The user inputs the values manually and is warned in case of values out of expected range (Supp Fig. S.7). ‘Calculate NEWS2’ button leads to a pop-up window with a calculator (Supp Fig. S.8). ‘Convert’ button refers to http://unitslab.com/. After pressing ‘Calculate’ the output is generated and presented to the user in the same window, below the ‘Patient Details’ field. Of note, during the experiment the participants did not directly interact with the interface. Static images with the tool’s output were provided.

In this study we used CORONET (COVID-19 Risk in ONcology Evaluation Tool [16]) which was developed to help determine the need to admit patients to hospital on the bases of their likelihood of needing oxygen (as generally oxygen is only given in hospital) and their severity of COVID-19, as indicated by predictions for required oxygen and/or death [37–39]. As a result, four key outcomes were established, arranged in a 0–3 point ordinal scale: discharged, admitted (≥ 24 h inpatient), admitted+O2 requirement (including ventilator support), and admitted+O2+died (with the death directly attributable to COVID-19 disease, not to cancer). These four outcomes were used as measures of disease severity. Contrary to an analysis of binary outcomes (e.g., need for oxygen vs. no need for oxygen), this strategy improved the ability to provide a complete clinical picture that was important for overall decision-making regarding hospital admission. During development, the model was first validated on an external cohort [16], and later on the most recent data from Omicron variant [40]. The CORONET tool is available online^1^ providing an interactive user interface with cross-device compatibility. The user interface is depicted in Fig. 2 (for more details please see Fig. S.7, S.8 and Suppl Methods 5).

#### Recommendation (R)

**Fig. 3:**
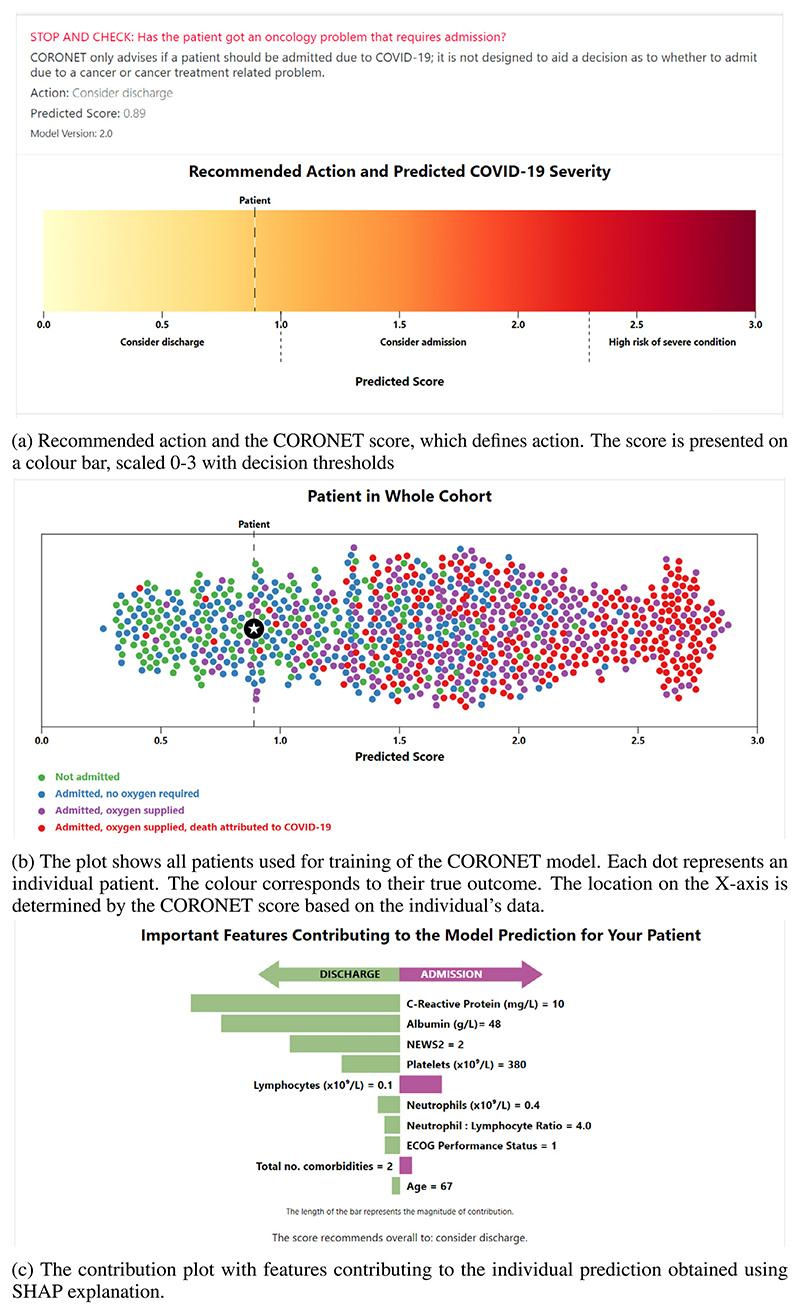
Output of the CORONET tool provided for each individual patient.

The basic output of the model is the recommended action and the CORONET score, which defines action. The score is presented on a colour bar, scaled 0-3 with decision thresholds (Fig. 3a). The score below 1.0 recommends discharge, above 1.0 admission, and above 2.3 suggests high risk of severe COVID-19 illness. Critically, this is in a group of patients who may also present to hospital with cancer or treatment related problems and not just COVID-19. This therefore adds complexity to the decision-making for the HCP. However, CORONET is only built to aid decisions regarding COVID-19 severity and requirement for admission. The CORONET model is a regression random forest, which was trained using the ordinal 0-1-2-3 scale as a dependent variable. True outcomes used as *y* are: 0-patient discharged; 1-patient admitted; 2-admitted and required supplemental oxygen; 3-admitted, required O2 and died. Thus, the model predicts the score in range 0-3 and the recommended action is delivered based on a pragmatically defined threshold [16,41,42].

#### Model explanation (Exp)

The explanation of the output consists of two visual components. Firstly, it shows a *scatter plot* (Fig. 3b) with the predicted score on the x axis for all patients used in model derivation (scores predicted in Leave-One-Out Cross Validation [16]). Each dot represents an individual patient and the colour corresponds to their true outcome. All patients are sorted from left to right according to their predicted score. The plot allows the user to locate the patient in question (marked by a star) in the whole cohort, considering both true outcomes and model’s recommendations. The point distribution elicits the model’s errors, i.e. some recommendations may be incorrect. For several ‘death outcomes’ the model predicted scores below admission threshold. Similarly, for some discharged patients the tool recommended admission. This reveals the performance of the model to the user.

Second, the explanation shows a bar plot with features contributing to the individual prediction (later referred to as *contribution plot*, Fig. 3c). The length of the bar represents the magnitude obtained using SHAP explanation [8]. The colour shows the direction of the contribution (towards discharge or admission). Features are ordered from top to bottom depending on the contribution. The figure serves as a local explanation of the model, where the bars change for each individual recommendation. The contribution plot is produced only for the patient in question, i.e. to see feature contribution for other cases the user needs to input new values. The contribution plot’s layout was iteratively derived from discussions between user experience designers and oncologists in order to produce the most intuitive visualisation. Among other considered layouts were: one side bar plot, waterfall plot [8], force plot [8], Partial Dependence plots [43] and Individual Conditional Expectation plots [44].

Overall, the model’s output consists of three cognitive chunks [28] organized in a three-step hierarchy: i) colour bar with the score; ii) scatter plot; iii) contribution plot. The derivation of the model and its associated source code are available at https://github.com/digital-ECMT/CORONET_tool.

### 2.3 Study design

**Fig. 4:**
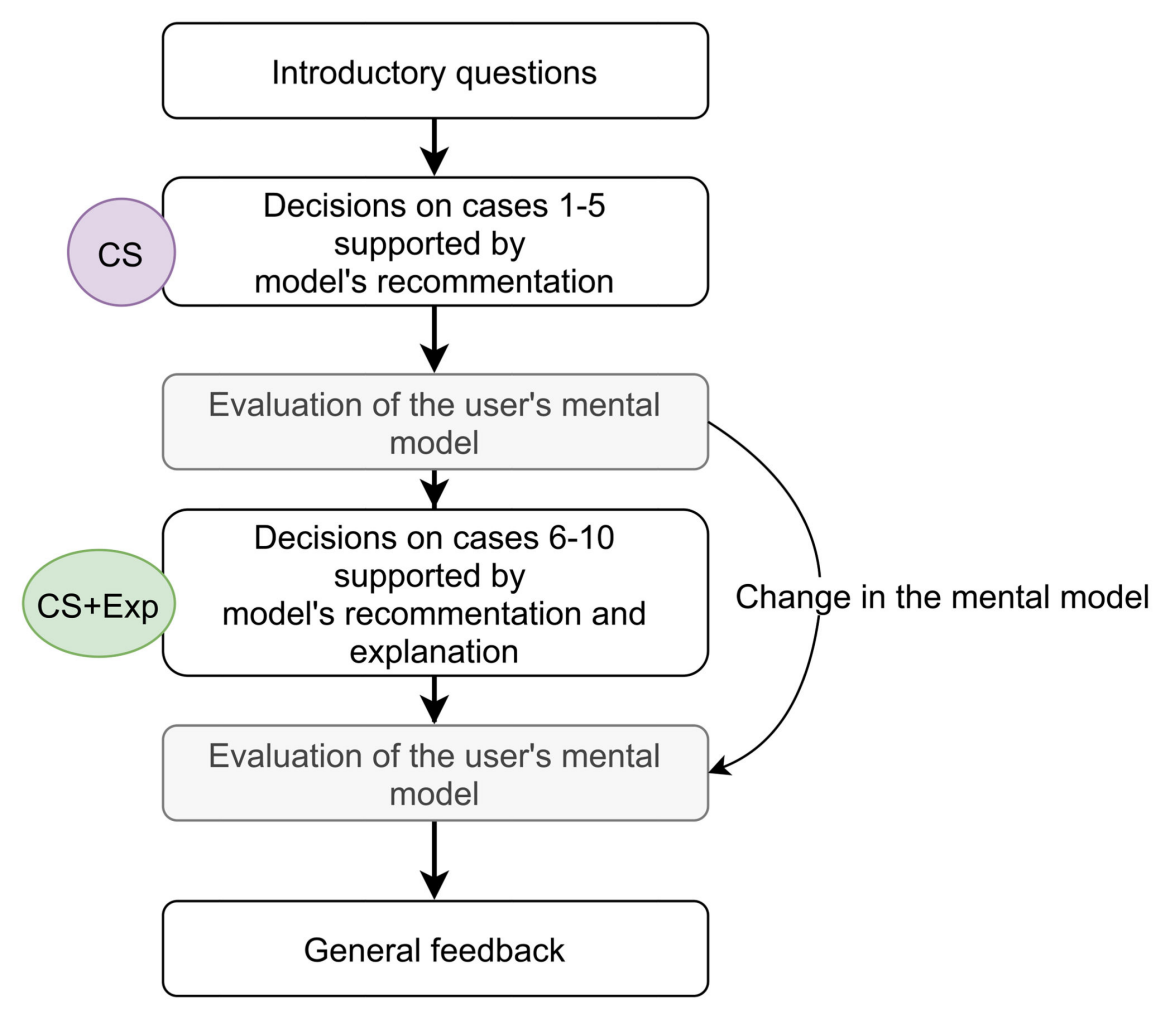
Experimental design. The within-subject design enabled examination of the change in attitude towards the model of each participant, as each of them was first supported by CORONET Score (CS) alone and then by CORONET Score and explanation (CS+Exp).

The study follows a within-subject design whereby each participant uses the tool in two conditions. Firstly, the participant is provided information in the form of a recommended action and a score on a scale of 0-3 (later referred to as **CS** - CORONET score), and told that the higher the patient’s score, the more severe the predicted outcome is. Secondly, the participant is then provided with the recommendation, the CORONET score and an explanation of how the tool has arrived at this score for the patient (later referred to as **CS+Exp** - CORONET score plus explanation). This approach minimises any random noise introduced by the variability on user’s clinical and technological exposure and previous experience. The order of presented cases was the same for all participants. The study design is depicted in the Fig. 4.

The online questionnaire consisted of six stages: i) introductory questions referring to the HCP’s background and expectations ii) five artificial patient cases with only the recommendation provided; iii) questions evaluating the model’s usefulness; iv) another five artificial cases this time provided with the recommendation and explanation; v) questions evaluating the model’s usefulness (same as in iii) vi) overall impressions of the tool. The questions were designed specifically for this study.

Ten artificial patient case scenarios were constructed, reviewed and approved by a senior oncology fellow and a consultant oncologist. The decision to admit or discharge was based on clinical guidelines and best practice. The scenarios were similarly structured and comprised an introduction to the patient (including demographics, presenting complaint, and relevant past medical history - all 10 cases are detailed in the supplementary material), the patient’s parameters from observations (vital signs), and blood test results.

Two of the ten patient cases (Daniel in CS, and Christine in CS+Exp scenarios) were intentionally built in a way that the model would wrongly discharge the patient, as CORONET considers features of COVID-19 severity only [37] and not oncological emergencies which might be concomitant e.g. neutropenic sepsis. In these cases, the healthcare professional should be able to apply their clinical judgement and recognise the patient would require admission for oncological reasons and subsequently override the model’s decision. In Daniel’s case, the admission is due to renal dysfunction and tumour lysis syndrome following chemotherapy received three days prior for his diagnosis of diffuse large B cell lymphoma. Therefore, this is a scenario where CORONET is thought to potentially ‘wrongly discharge’ the patient as it does not take into consideration oncological emergencies as part of its recommendation. In Christine’s case the admission is due to her recent chemotherapy course. Again, because CORONET does not take other types of infections and treatment related presentations, this is a situation where CORONET is not adequate in making an accurate decision.

These cases were included to assess for over-reliance on the model (automation bias). We analysed the concordance between clinicians’ decisions, and the model’s recommendations and the correct actions approved by our team of experts.

We tracked the time spent on each part of the questionnaire ensuring that it was not quickly skipped when the user lost interest or was engaged with external tasks. It also allowed for tracking the time spent on decision of each patient case.

### 2.4 Selection of participants

This research project was ethically approved by the research ethics committee [REC reference: 20/WA/0269]. Participants were required to be clinically active healthcare professionals (determined as having patient contact in at least 10% of their working hours) and currently working with patients with cancer presenting with COVID-19. This included senior doctors, junior doctors, physician associates, pharmacists, advanced practitioner nurses, and staff nurses. Any participants who failed to meet these inclusion criteria were excluded. Participants were recruited via email invitations to NHS Trusts, clinician networks, and social media groups. Although CORONET was already available online by the time of the experiment, to the best of our knowledge the participants were not using the tool prior to the experiment. The familiarity with the tool and/or with the preprint [41] was not required. Knowledge related to ML, computational methods or statistics was not required.

## 3 Results

### 3.1 Participants

23 healthcare professionals participated in the experiment, with various levels of experience and expertise (Table 1). The median knowledge regarding managing patients with COVID-19 was five (in one to seven scale), evaluated via direct question. Most of the HCPs felt very comfortable using new technologies. 35% of participants perform statistical analysis at work, and majority of participants do use online calculators or scoring systems, similar to CORONET (83% and 65%, more in Supp Table S.1).

### 3.2 HCPs want to know both contributing features and uncertainty. Uncertainty was considered to be more important than contributing features

**Fig. 5:**
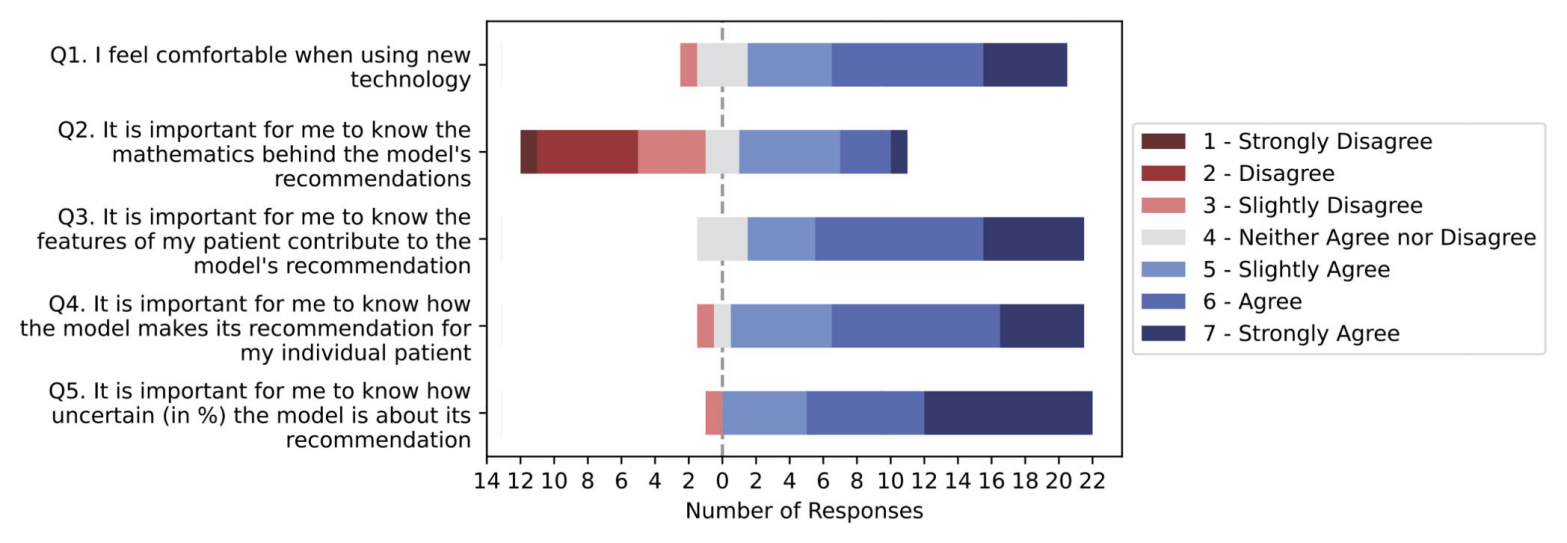
Responses to questions related to HCP’s expectations regarding a ML model. Q3-Q5 relate directly to the expectation regarding model transparency and explanation. Q3 investigates the need for global explanation, and Q4 asks about local explanation for the “individual patient”.

The responses regarding the expectations for the ML-based DSS are depicted in Fig. 5. 87% (20/23) of HCPs were interested in knowing the features contributing to the model’s recommendation, three HCPs had neutral opinions. 91% (21/23) considered the model’s explanation of an individual recommendation as important. Apart from knowing *why* the model recommends such action, the uncertainty behind the recommendation was even more essential for the HCPs: 96% (22/23) of them at least slightly agreed, and 43% (10/23) strongly agreed, which is the highest ‘strongly agree’ proportion in the study.

Knowing the mathematical framework behind the model is significantly less important than the local/global explanation and the model’s uncertainty (*p* < 0.001, Kruskal-Wallis test, Supp Table S.5). Intriguingly, 73% of HCPs who were not interested in the mathematics behind the model would like to see an associated model explanation and uncertainty. This points into largely unaddressed research questions on the dialogue between explanations and safety properties (such as a representation for uncertainty) as proxies for risk assessment.

### 3.3 Visual explanations were easy to interpret

**Fig. 6:**
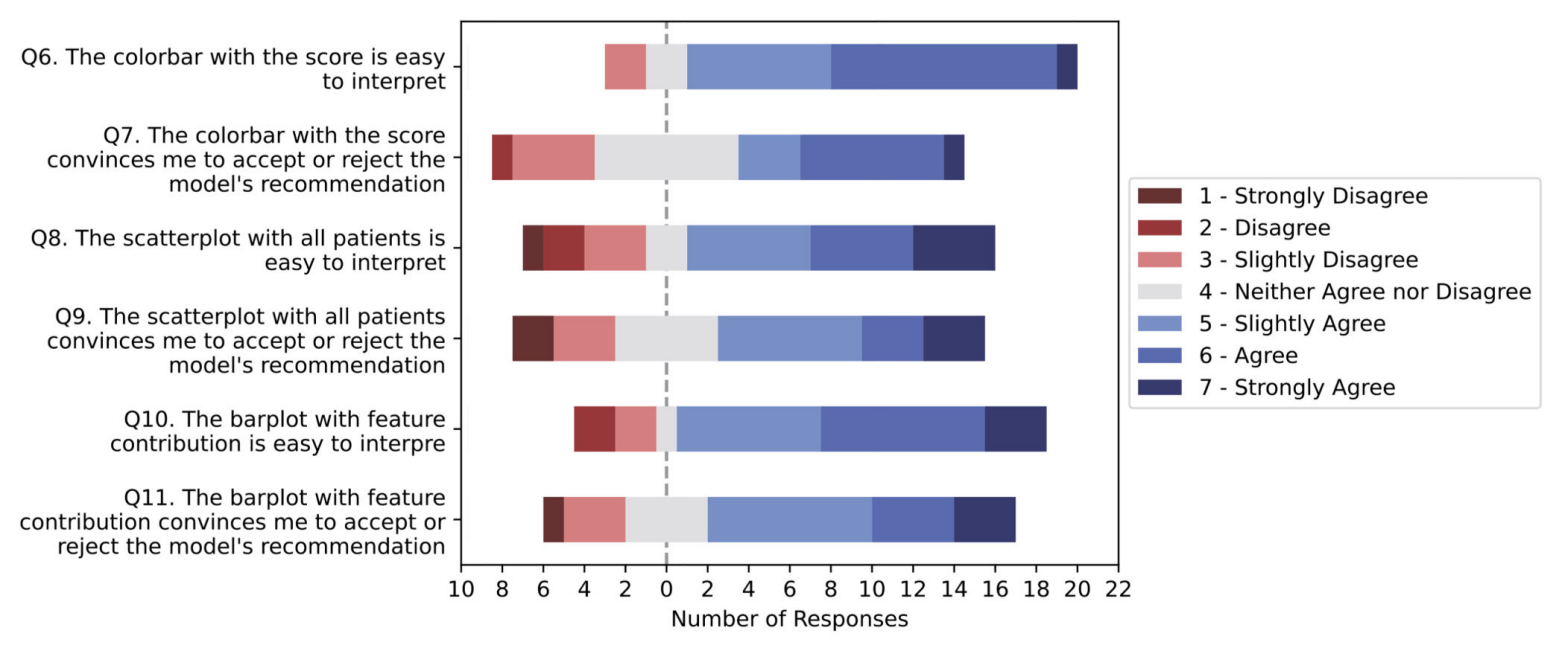
Responses related to HCP’s easiness of interpretation of visual output of CORONET and whether it convinces the user to accept the model’s recommendation.

Explanation visualisations were easy to interpret and contributed to convincing HCPs to accept or reject the model’s recommendation (Fig. 6, Supp Table S.2). This aspect was measured via direct questions for each visualisation: i) whether it was easy to interpret and ii) it was convincing to accept of reject the recommendation. For the majority of HCPs, the colour bar was easy to interpret (83%; 19/23). Interestingly, the CS (score without further explanation) presented on 0-3 colour scale convinces 48% (11/23) HCPs to accept/reject the recommendation. Among these 11 convinced by CS, the average response to questions regarding expectations are high (>5.7).

We found no significant difference between the ease of interpretation of the colour bar, scatterplot or contribution plot, as well as no difference in their persuasive power (*p* > 0.05, Supp Table S.3). We did not find any significant correlation between questions related to the visual output and either knowledge on the management of patients with cancer who have developed COVID-19, nor to expectations for ML-based DSS (Supp Table S.4). Thus, the perception of the visual output was not affected by expectations, nor the competence in the task.

### 3.4 Explanations did not significantly impact on the decision-making

**Fig. 7:**
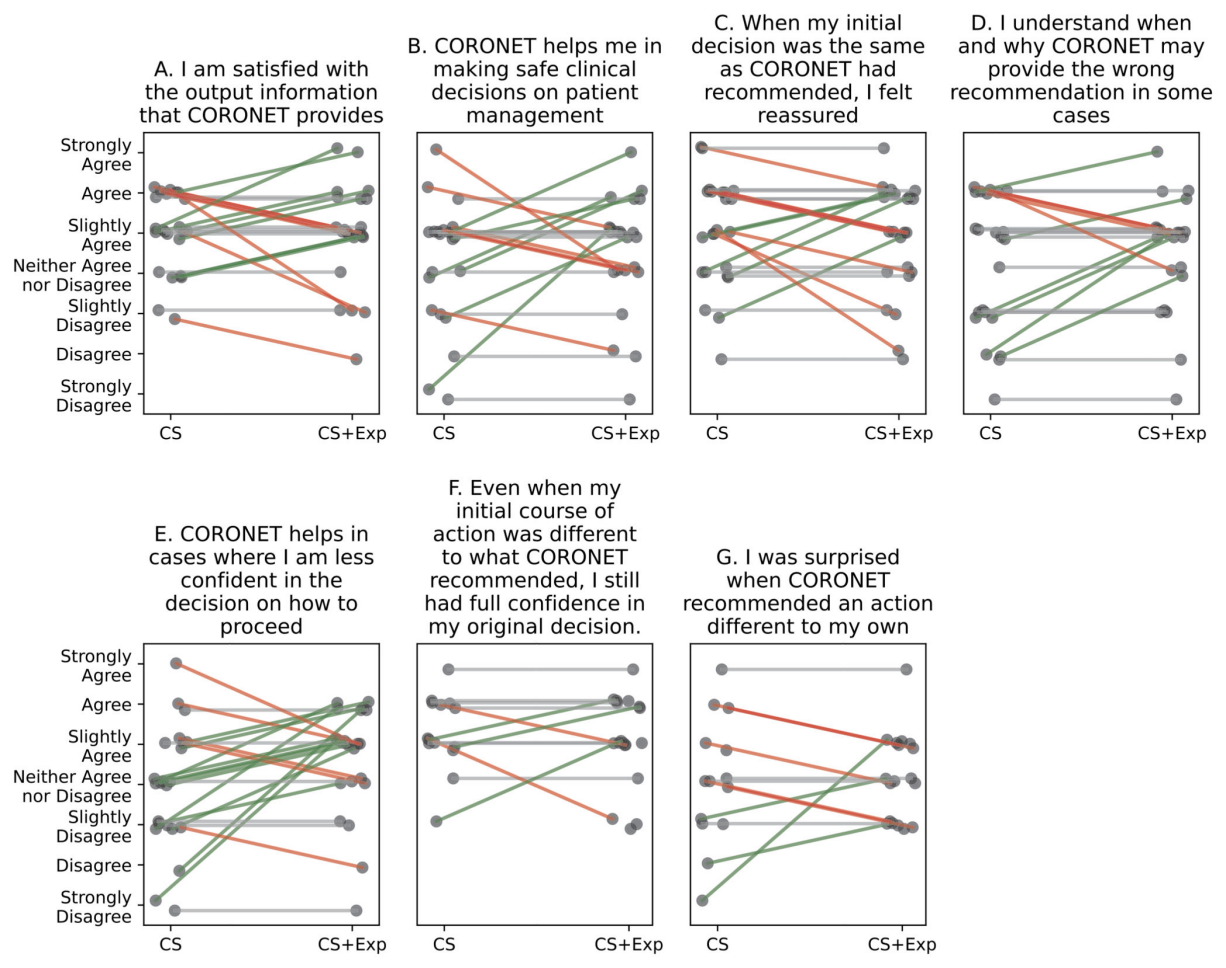
Paired answers for the same questions among 23 Healthcare Professionals. Each dot is an individual answer. Lines: grey - no change; green - positive change up in the Likert scale; red - negative change. No statistically significant change observed in pairwise comparison.

In the pairwise comparison of responses between CS and CS+Exp scenarios, we did not find a statistically significant change in the HCPs’ attitude towards the model (i.a. satisfaction, trust, understanding, reassurance). The results are summarized in Fig. 7, Fig. S.1, Supp Table S.6.

The explanation did not improve satisfaction (RQ2, Fig. 7 question A), trust (RQ3, Fig. 7 questions B, C, E) nor the understanding of how the model produced the recommendation (RQ1, Fig. 7 questions D, F, G). Of note, we observe a slight but not significant positive change (*p* = 0.056) in ‘help in cases where I am less confident in the decision on how to proceed’.

The majority of HCPs replied positively (57-74% answers at least ‘Slightly agree’ in quest. A-C) regarding the CS output alone. We established the following associations between positive responses and HCPs’ expertise and expectations:

- The lower the expertise, the more helpful the tool appeared to be, even when no explanation is provided (*r* = −0.482, *p* = 0.02, Fig. S.2).
- The higher the need for knowing the contributing features, the less helpful the CS output is likely to be: in making safe decisions (*r* = −0.653; *p* = 0.001, Fig. S.3) and in cases where HCPs are less confident (*r* = −0.553, *p* = 0.006, Fig. S.3).

Additionally, being convinced by the colour bar was correlated with satisfaction and reassurance in the CS only scenario (*r* = 0.484, *p* = 0.019 and *r* = 0.527, *p* = 0.01, Fig. S.4).

### 3.5 Model explanations may have an adverse effect on HCPs

CS-Exp output of the model led to both positive and negative changes in satisfaction, helpfulness of the model, reassurance and understanding. 34% HCPs were more satisfied with CS+Exp than CS, but in 23% their satisfaction decreased (RQ2).

Higher satisfaction was weakly correlated with ease in the interpretation of the contribution plot (*r* = 0.436, *p* = 0.038), but not correlated with being convinced by it (*p* = 0.24). HCPs who were convinced by the contribution plot in the CS+Exp scenario (65%; 15/23), showed high levels of satisfaction both without (average 5.4) and with an explanation (average 5.6). This is also true regarding the scatter plot.

26% of HCPs said that the tool with CS+Exp was more helpful in making safe clinical decisions, with 26% stating the opposite. There was a positive correlation (*r* = 0.63, *p* 0.001, *p_adj_* = 0.135) between change in satisfaction and change in help in making safe decisions. 22% of HCPs felt more reassured in the CS+Exp scenario, while 35% felt the opposite.

The provided explanations did not change the level of understanding of when and why the model produces wrong recommendations for 57% (13/23) of HCPs (RQ1). For 17% the explanation even led to lower understanding. This cannot be attributed to the time spent on the patients’ analyses, nor ease in interpreting the diagrams (*p* > 0.05).

The change in understanding wrong recommendations was associated with the change in the reassuring utility of the tool (*r* = 0.762, *p* < 0.001, *p_adj_* = 0.003), which is the strongest correlation found between changes (Supp Table S.7).

The highest percentage - 52% of positive changes was observed to help in cases where the HCP is less confident. Among them, there were three HCPs who rated their knowledge of managing patients with COVID-19 as 2 (RQ5). However, 22% found CS+Exp less helpful.

### 3.6 Over-reliance on the model’s recommendation leads to wrong decisions

**Fig. 8:**
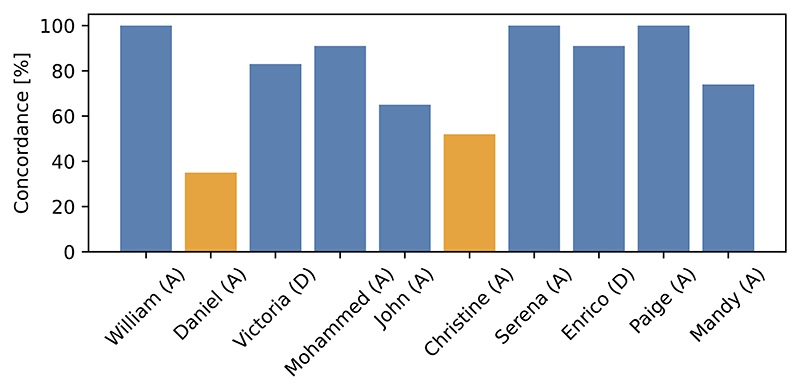
Concordance between decisions made by respondents and correct action approved by our team. Higher is better. (A) - correct decision ‘admit’, (D) - ‘discharge’. Daniel and Christine (orange bars) were cases where CORONET recommends incorrect discharge.

There are only three cases where all the HCPs made the correct decision, and six cases where the concordance with the correct decisions was more than 80% (Fig. 8). Of note, the concordance does not depend on whether a patient should be admitted or not when the tool recommends correctly (on average 88% vs 87%, *p* = 0.73).

However, the lowest concordance with the correct decision was observed for cases where CORONET recommended the wrong action (RQ6, Fig. 8). In Daniel’s case 65% (15/23) HCPs decided to discharge, as CORONET recommended, while for Christine this accounted for 48% (11/23). As the level of complexity and difficulty in decisions across the cases 1-10 was similar, we argue that the lowest concordance for Daniel and Christine is caused by the wrong recommendation provided by the model. The results strongly suggest that for both types of output (CS and CS+Ex) HCPs over-relied in the recommendation provided by the model.

### 3.7 Quicker decisions due to over-reliance on explanations

Decisions which were in agreement with the model recommendation required less time (RQ6, Table 2, Fig. S.5). For cases 1-5, where the tool provides only the CS, the median time spent on one case was 48s when the user agreed with the tool, and 61s otherwise. For cases 6-10, where the CS+Exp was provided, we observed a statistically significant difference: decisions required 38s for decisions aligned with and 84s (median values) for decisions opposed to the tool’s recommendation (*p* < 0.001, *CLES* = 0.754, MWU test).

As described in the previous section, some respondents needed less time when deciding on cases where the explanation was delivered. Further investigation of the agreement with the model reveals that when the HCP agrees with the recommendation (RQ7), the combined CS+Exp output leads to significantly quicker decisions than CS output: 38 s for CS+Exp and 48 s for CS (*p* = 0.04, *CLES* = 0.59). Moreover, when the explanation is provided, it takes more time to decide when the model contradicts the user’s decision, but the difference to CS is not significant. Median time for CS output was 61 s, compared to 84 s for CS+Exp (*p* = 0.17, MWU test).

### 3.8 Positive feedback, but no attribution to the explanatory model

**Fig. 9:**
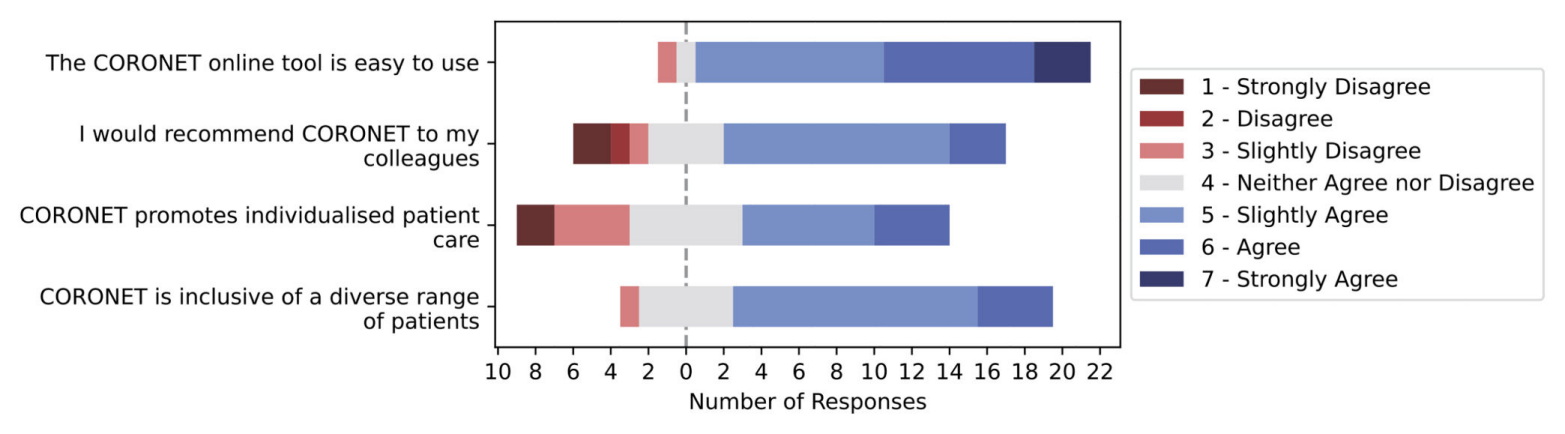
Positive feedback regarding the CORONET evaluated at the end of the experiment.

General feedback regarding the CORONET model is positive (Fig. 9). The model and supporting interface is considered easy to use, and the majority of respondents would recommend the tool to their colleagues.

**Fig. 10:**
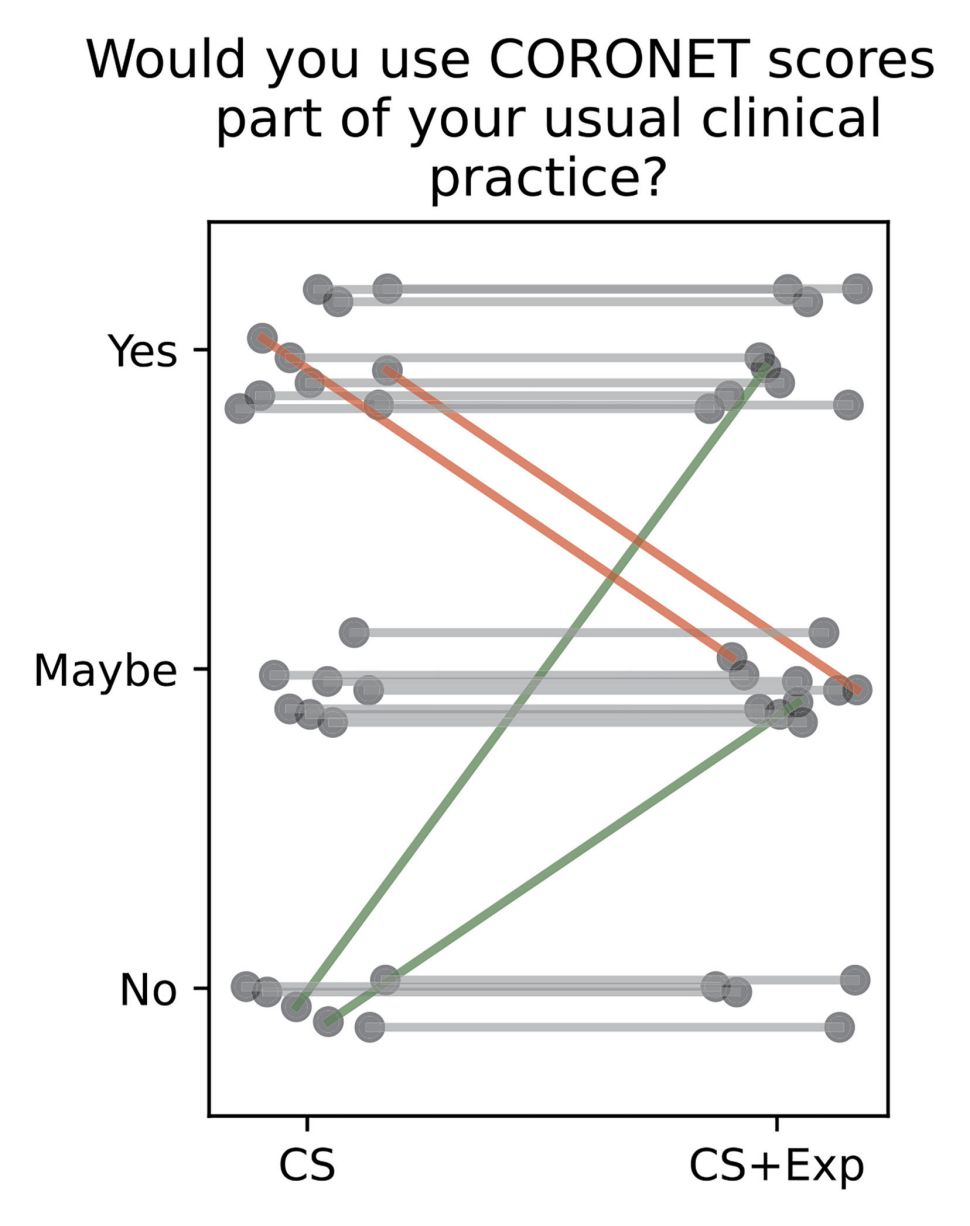
Evaluation of clinical utility of the tool. Same question was asked twice, for CS and CS+Ex. No significant change (*p* > 0.05).

We found no impact of the explanatory component on the clinical utility of the model (Fig. 10), as the explanation did not improve the attitude towards whether to use the CORONET in clinical practice or not (RQ5, *p* > 0.05, Wilcoxon signed-rank test).

### 3.9 Respondents’ engagement

**Fig. 11:**
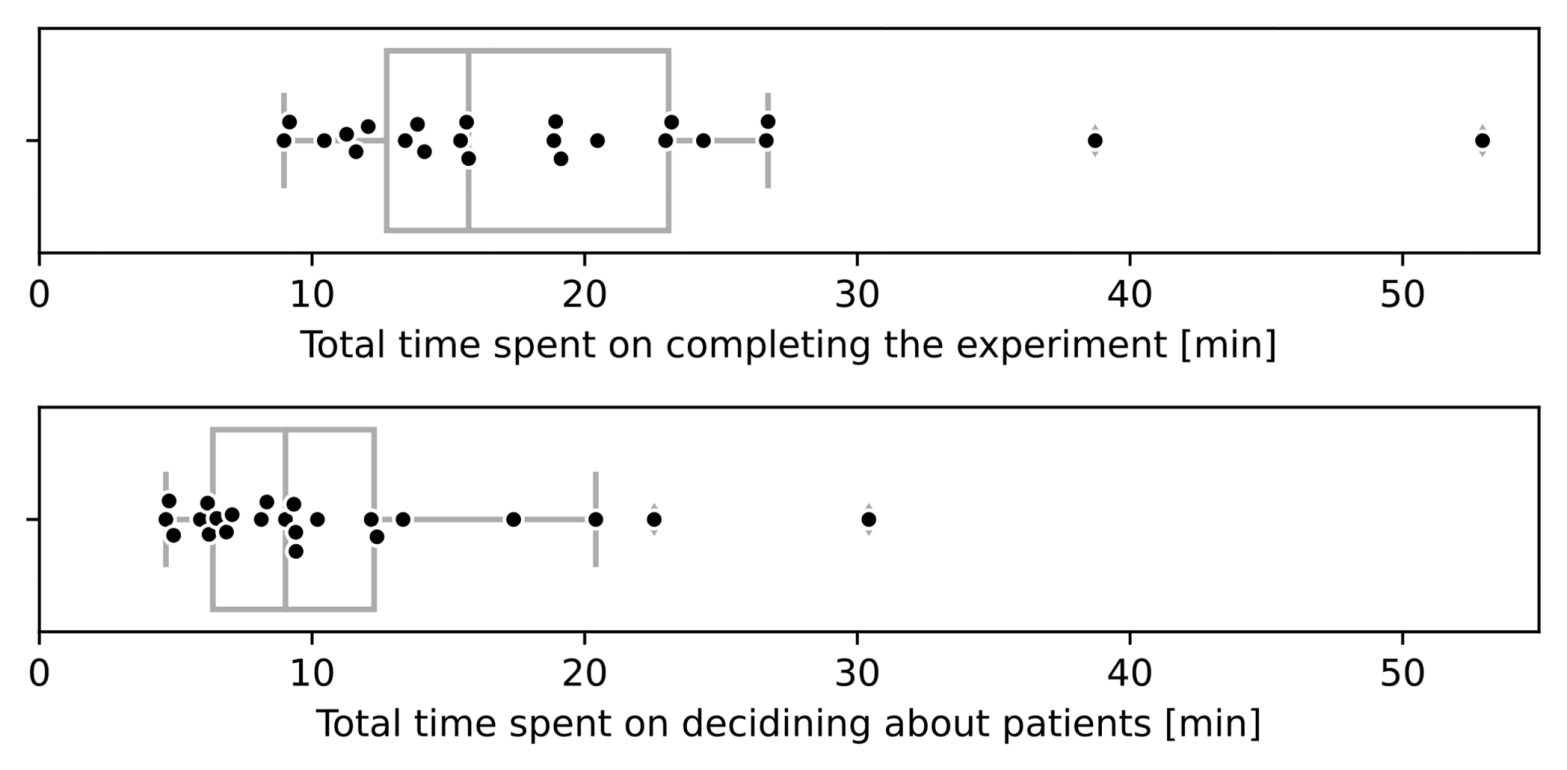
Total time spent by 23 Healthcare Professionals.

Our experiment is supported by an online questionnaire, which is to be completed without direct supervision and time limits. Although the questionnaire is estimated to require ≈ 30min, the actual average completion time was ≈ 19min (median 16 min, Fig. 11). Of note, nine HCPs completed it in less than 15 min, spending less than six min on deciding about 10 patients. We also identified several anomalies in the time spent in particular sections which we manually curated (see details in supplementary material). Most likely they were caused by occasional interruptions, which are inherent in the experiment conducted in the clinical setting, where the respondents may be distracted by more urgent matters.

Overall, we did not find a significant difference between total time spent on deciding on patient cases between CS and CS+Exp (*p* = 0.846, Wilcoxon signed-rank test, Fig. S.6). However, 56% (13/23) HCPs spent less time when CS+Exp was provided. We did not find any correlation between this decrease and other aspects investigated in previous paragraphs.

## 4 Discussion: pragmatic clinical embedding of explainable ML models

### CORONET explanation among the existing taxonomies

The empirical analysis conducted in this study fits into a *domain expert experiment with the exact application task*, which is an application-grounded evaluation, with real humans and real tasks (according to the taxonomy introduced in [28]).

In designing the CORONET output, we followed the *perceptive interpretability* framework [45], emphasising an immediate interpretation: *you see, and you know*. We argue that the contribution plot follows this assumption.

According to taxonomies established in [5] and [19], the output of the tool investigated in this study delivers:

- **modularity:** each interpretable element of the output (cognitive chunk) appears as a separate visual component. In the contribution plot, each bar delivers a meaningful portion of information and can be interpreted independently (e.g. low contribution of albumin level to recommendation of ‘admission’)
- **relevance:** it provides insight for a particular audience, in this case clinicians seeking a patient’s characteristics critical for the decision (contribution plot), and also how the patient in question can be located in the whole cohort (scatter plot). Additionally, the colour bar and score describe how severe the patient’s outcome is expected to be.

However, CORONET’s output does not deliver:

- **simulatability:** a model is simulatable when the user can reason and simulate how the model produces the output for an arbitrary input. It could be possible (assuming limited complexity of the model) for algorithms such decision tree or a set of rules. However, as CORONET uses a Random Forest regression model, with multiple (>100) decision trees used to arrive at the prediction, the user cannot precisely elicit the formal decision process.
- **unambiguity:** CORONET provides local explanations, focusing on individualised patient care. Based on that, it is not possible to reveal how the model behaves in various parts of the feature space. Such behaviour was investigated during model derivation [16] with the support of dependency plots. However, these are not presented to the user in the context of this study.

Analogously to unambiguity, the user is not able to evaluate the *descriptive accuracy* [5], which measures how well the relationship learned by the model is reflected in the explanation. Such evaluation was performed during model development, and then intentionally excluded from the cognitive chunks presented to the user. We argue that additional cognitive load would hamper the benefit of such information [46,24].

### Characterising HCPs’ attitude on the explanations

More than 87% of participants stated that knowing the contribution of features is important, both for the overall model and individual recommendations. The most important aspect, with the highest number of ‘strongly agree’ answers, is to know the model’s uncertainty. This could suggest that HCPs, are self-reportedly more comfortable in assessing risks and uncertainty, rather than interpreting the feature contribution to the output of the ML model. Hence, delivering a model’s uncertainty may contribute more to building trust in comparison to a break-down explanation of the features’ contributions. However, this conclusion is solely based on the proportion of ‘strongly Agree’, as we did not find significant differences between expectations regarding uncertainty, feature contribution and individual recommendation (p>0.05, Kruskal-Wallis test, Supp Table S.5). An intuition on the mathematical principles behind the model is perceived as significantly less important (p<0.05).

Showing uncertainty increases trust and the likelihood of following the model prediction [26], under a low cognitive load setting [29]. Limited cognitive investment in the model interpretation would result in a reduced understanding of the output. While the model used in our study does not explicitly provide uncertainty of its prediction, it recommends a binary action based on a numeric score and a threshold, which are shown to the HCPs. We argue it serves for the user as a proxy of the model’s recommendation confidence.

### Unmet need to build pragmatically grounded explanatory models

Although the majority of HCPs (78%; 18/23) found the explanation component accounting for the features’ contributions easy to interpret, still 17% of HCPs found the explanation of the model difficult (RQ2). This points to opportunities for the design and optimisation of explanatory models from the point of view of end-user interaction. Contemporary explanation methods such as SHAP or LIME focus on the calculation of a faithful explanation for the model, abstracting away the pragmatic aspects of this explanation (e.g. their interaction with domain experts). Recent work binding representation design and cognitive models [47,48] may provide a formal avenue for designing more pragmatically efficient explanatory models.

Explanations in the form of feature importance may act as a safety feature, drawing the user’s attention to features omitted by initial judgement but marked as highly relevant by the model [49]. According to [50] the feature-based explanatory models can be in one of three groups: desired (expected by included in the model), ambivalent (user is indifferent about whether they are included or not) or prohibited features (expected to be omitted, following ethical ML principles). Displaying contributing features allows the user to verify whether the model utilises expected features instead of inconsistent or irrelevant features, based on supporting domain knowledge. In our study, we asked the HCPs explicitly whether the model included all desired features. Additionally, no prohibited features were recognized in the model. One of the downsides of feature importance is that it may lead to confirmation bias, both in the model derivation phase [51] and in the post-hoc interpretation of the output. Practically, it is caused by noticing only the features that confirm previous assumptions and overlooking other, potentially incorrect features.

### Ambiguity on the utility and perceived value of explanations

A considerable number of HCPs did not change or even had a worse attitude towards the model after the explanation was provided (RQ1). 57% of them reported the same level of understanding, and 17% were understanding even less why the model provides recommendations different to their own. Satisfaction remained the same for 43%, while 23% were less satisfied with the explanation. A possible explanation is an information overload (cognitive effort) effect or simply not meeting the expectations regarding the explanation model.

For 48% and 26% of HCPs the model with and without explanation was considered the same in terms of support for making safe clinical decisions and helping in ambiguous cases, respectively. For 26% and 22% the explanation even decreased the helpfulness. As high as 35% felt less reassured when model provided the same recommendation as initial decision. We hypothesise, that the explanation could introduce further uncertainty in the clinical judgement, as it may have differed from the clinical assessment in particular cases. This highlights previous adoption barriers towards ML-based decision support tools, i.e. lack of agreement with the model, lack of knowledge of the model, HCPs’ attitude toward the model [36,52,53]. Despite the widespread notion on the need for safe and explainable models, we found in our study that a step towards such models remained unnoticed or negatively perceived among almost half of the participants (RQ3). As suggested in [53], initial scepticism regarding ML-based models may have caused such a rejection.

This implies that explainable ML researchers should further move from an algorithm-centric perspective [45], to a pragmatic perspective, i.e. prioritising their pragmatic embedding within the experts’ decision-making process. Feature contribution, well-received in the data science community, is not necessarily acknowledged among HCPs. Intensifying the dialogue and creating feedback loops between explainable AI researchers and domain experts will be an essential part of the development of pragmatically relevant explainable ML models.

Although explainable ML is advocated as the methodological silver bullet for addressing transparency issues [1], some user studies, similarly to this paper, show evidence of a limited or even no impact of the explanation on performance in a task [54,18,55–57]. This highlights the direction of further research in what explanation is expected depending on the context, e.g. clinical setting. Our study shows that we should be careful about our preconceived notions about what needs to be explained.

### For many HCPs, explanations did not deliver a critical understanding of the model

Explanations did not improve the critical understanding on why the model recommends a different action to the HCPs for 57% of the participants (RQ4). One possible reason may be a misunderstanding of the difference between local and global explanations. The barplot used to visualise feature importance shows the magnitude of the contribution of each feature to the recommendation for an individual patient. For each analysed case the bars can be reordered. This may lead to an extra complexity in interpretation and shows the simplistic design of standard explanation visualisation devices. However, when promoting more individualised patient care one should also expect higher variation in local explanations of the model. Second, the tool is oversimplifying the complexity of the patient and does not refer to the underlying deep biological processes which may explain a biomarker and its relation to the recommended action. The tool is focused on one aspect of the patient (in this scenario COVID-19) and a finite set of predictors. This is often overlooked by the HCPs, and the limitations of the model remained unidentified even with the explanation provided.

### Explainable models help HCPs to address ambiguous cases

The highest number of positive changes between CS and CS+Exp was observed to support clinical cases where HCPs were less confident in their decisions (RQ5). Interestingly, less experienced HCPs tended to trust the model more. This confirms the findings of [58] and [59] where less experienced clinicians are more likely to rely on the recommendation, changing their initial decision. This suggests that the main function of a ML-based (data-driven) clinical decision support system, such as CORONET, is to be helpful when the user is uncertain, pragmatically positioning the model within the clinical workflow [60]. This is aligned with the direct feedback received from HCPs (see Supp 5). The recommended action, together with the explanation, may deliver new evidence and justifications for more ambiguous cases.

### Explainable models help HCPs to acquire new domain knowledge

When the user still lacks a consolidated domain prior knowledge, the explanation may act as a guideline or a source of new evidence-based knowledge, pointing into a second function of explainable ML models (RQ5). In [20] explanations only had an impact on HCPs who felt they had insufficient knowledge to achieve their given task, which may signify that the knowledge gap was addressed by the model’s output.

### Quicker decisions for explanatory models in contrast to black-box models

57% (13/23) of HCPs spent less time on decisions for cases 6-10 (the cases with explanations), when more information was delivered to end-users (RQ6). Potential reasons are: i) lost interest in the experiment; ii) gained fluency in interpreting the provided information. After the first five cases, HCPs familiarized with the layout of the decryption were able to decide on upcoming cases quicker; iii) provided explanations expedited the decision due to over-reliance on the tool. The questionnaire was not designed to verify points i) and ii) leaving this point of ambiguity. Cases 6-10 reflect the same level of difficulty from cases 1-5.

### Models and explanations can increase confirmation bias

The results suggest that when the recommendation agrees with their initial decision, HCPs decide quicker, when compared to a model disagreement setting (RQ6). Furthermore, when explanations are provided, the decision is comparatively quicker. When the tool contradicts the clinician, explanations lead to a longer reflection on the decision. This highlights the risk of confirmation bias, possibly caused by the explanation that increases the reassurance on the decision (RQ7). This aligns to the results in [1] where users tend to use the explanation to support the justification of their prior decision. The authors found that users reinforced pre-existing beliefs when the explanation supported them but did not abandon them when it was the opposite. Of note, model accuracy also affects the likelihood of adjusting prior beliefs. The risk of confirmation bias is particularly high when the explanation closely matches HCP’s expectations [53]. In some cases, the explanation may appear reassuring even if it does not explain the model [61].

### Evidence of explanations reducing automation bias

On the other hand, explanations drove users to reflect longer on the decision when disagreeing with the model outcome (RQ6). More time may indicate cognitive forcing, which is reported to reduce over-reliance on the model [24]. At the same time, to reduce the automation bias, the explanation must reduce the cognitive load [46,34]. Working memory constraints, together with increased cognitive load, lead to higher uncertainty in decision-making processes, thus increasing automation bias. This is particularly true in clinical settings where multiple contextual factors affect the reasoning process of HCPs [27].

### Over-reliance on the model’s recommendation

Some evidence in the literature suggests models that use explainability techniques can hamper the user’s ability to detect when a serious error is made [56]. The explanation may excessively increase users’ confidence in an algorithmic decision, communicating a false impression of correctness and rigour and resulting in decreased vigilance and auditing of the output [62,63]. In addition, [33] and [64] reported higher error rates when decisions relied too heavily on automation, resulting in bias towards a wrong action despite, evidence available which should have resulted in the user overriding the model. Automation bias is also linked to higher cognitive load and to consistently higher accuracy of the model [46], and familiarity with the task [20]. In our study, we identified over-reliance on the model for the cases where CORONET was intentionally wrong (RQ6). For these incorrect recommendations, the HCP should have overridden the decision and admitted the patient. Only 35% (CS scenario) and 52% (CS+Ex) HCPs took that critical perspective over the model.

### 4.1 Assumptions & limitations of this study

#### General concordance among clinical judgement

In the assessment of AI models for decision support in healthcare, a well-known challenge is the definition of a ground truth [65], as decisions may vary across HCPs. Thus, a panel of HCPs is recommended rather than a small number of annotators. In our study, the ‘correct’ actions were defined by a panel of two experienced oncologists.

#### Lack of controlled environment

An ideal environment for HCPs to participate would be at a face-to-face setting. Due to the pandemic and social distancing measures, the study was developed in an online setup. In this context, devices used by individual HCPs could not be controlled, which may have impacted on the consistency and the quantity of the usability feedback on each type of device. Despite this, the developers of CORONET designed its interface for cross-device compatibility (e.g. phones, tablets, and computers), and therefore by undertaking this method of design, we were able to capture feedback on CORONET’s usability based on a mixture of device types. Due to remote setting and lack of control, potential additional factors may have impacted the results. However, the experimental design assumes performing a task that HCPs are familiarized with: admit or discharge a patient based on a detailed clinical description. Thus we argue that all participants had very good understanding of the task.

#### Rush in completing the questionnaire

We argue that attempting to understand the model and therefore building trust in the tool require a substantial time commitment for some HCPs. Designing a study which is supervised, better controlled, and which better incentivises HCPs for a longer time commitment would improve the current study design.

#### Use of simulated patient cases

To preserve the confidentiality of the patients, artificial patient case scenarios were used instead of real-life data. The cases were constructed in a clinically consistent manner by two domain experts. However, this does not take away the fact that in real life, a HCP would undertake clinical observations of the patient and account for any visible signs of distress and illness, such as any skin changes, unusual responses, and patient examination findings, which is not possible to reproduce in an artificial setting.

## 5 Conclusions

In addition to the acceptability of the predictive model [32,66], a pragmatic evaluation of the user-model interaction is key to a successful deployment of ML-based recommendation tools in healthcare. The deployment depends not only on a explanation-interfaces [22] designed by software developers but to a greater extent, as shown in this study, on clinical performance evaluated by biomedical experts, initial attitudes and biases. This paper contributes a pragmatic evaluation framework for explainable ML models for clinical decision support. The study used a within-subject design involving 23 healthcare professionals and compared an explainable ML model with a black-box model. Such a relatively small number of participants and their heterogeneity (i.e. various backgrounds and levels of expertise) may hinder drawing statistically robust conclusions. However, we argue that the study points into the direction of a more nuanced role of ML explanation models when these are pragmatically embedded in the clinical context and the results settle a solid base for further research. HCPs acknowledged the role of explanations as a safety and trust mechanism. Communicating the uncertainty behind the model emerged as a stronger requirement, when compared to the explanations.

Despite the general positive attitude towards explanations, for a significant set of participants (17-35%), an undesirable effect for the explanations was observed, possibly due to an increase in cognitive effort. Moreover, explanations did not improve the critical understanding of the model for 57% of the participants (i.e. their ability to detect an error in the model). We also found that explanations can increase confirmation bias, possibly accentuating over-reliance in the model. On the other hand, explanations drove HCPs to reflect longer on the decision when disagreeing with the model outcome, evidencing explanations as a possible mechanism for reducing automation bias. There was strong evidence that explainable models better supported HCPs to address ambiguous clinical cases (cases where HCPs were not certain about their decision). Also, explainable models helped less experienced HCPs to acquire new domain knowledge.

This work points to still open research questions in the area, including the need for further pragmatic evaluation of explainable models in complex clinical workflows, the co-development of models of explanations with domain experts and a better understanding of the dialogue between other model safety mechanisms and explanations.

## Supplementary Material

Supplementary material related to this article can be found online at https://doi.org/10.1016/j.artint.2022.103839.

upplementary Information

## Figures and Tables

**Table 1:** Characteristics of 23 healthcare professionals participated in the experiment.

Age group	n
21-30	6
31-40	10
41-50	3
51-60	4
	**n**
Advanced Nurse Practitioner or higher	2
Consultant / attending physician	5
Doctor (within 2-4 years of graduating / specialty trainee (ST) / fellow	2
Doctor within first year of graduating (FY1) / intern	1
General practitioner / GPST / community doctor	3
Pharmacist	1
Registered Nurse	1
Specialist Nurse	1
Specialist registrar / ST 3+ / senior resident/senior fellow	7
	**Median [min,max]**
How comfortable you feel when using new technology?	6 [3,7]
Knowledge on the management of patients with cancer who have developed COVID-19	5 [2,7]

**Table 2:** Time spent on individual decisions for cases 1-5 (CS) and 6-10 (CS+Ex). p values from Mann Whitney-U Test; significance: ns - non significant, *p* > 0.05, * - *p* < 0.05, ** - *p* < 0.01, *** - *p* < 0.001.

Time [s] median [Q1,Q3]	Action the same as the recommendation	Action against the recommendation	p	Significance
Cases 1-5, output: CS	48 [34,74]	61 [39,85]	0.097	ns
Cases 6-10, output: CS+Exp	38 [22,64]	84 [50,116]	0.000	***
^p^	0.042	0.170		
Significance	*	ns		

## Data Availability

Data will be made available on request.

## Footnotes

1 https://coronet.manchester.ac.uk.
