# Supplementary material for: Assessing the communication gap between AI models and healthcare professionals: Explainability, utility and trust in AI-driven clinical decision-making
Source: Artif Intell. Author manuscript; Available in PMC 2026 Jan 16. (PMC7618637; doi:10.1016/j.artint.2022.103839)
Supplement: upplementary Information [file EMS212064-supplement-upplementary_Information.pdf]

Supplementary Information

Supplementary Methods

List of questions

Below we list all questions from the experiment, that are relevant in the analysis aiming to answer broader research questions from the previous section. The questions were produced by both computer scientists and oncologists, same as involved in designing clinical cases.

Questions related to demographics:

- D1. What is your job title?
- D2. What is your age group?
- D3. Which of the following tasks do you perform on computers at work?
- D4. Rate your knowledge on the management of patients with cancer who have developed COVID-19

Questions related to expectations:

- Q1. I feel comfortable when using new technology
- Q2. It is important for me to know the mathematics behind the model's recommendations
- Q3. It is important for me to know the features of my patient contribute to the model's recommendation
- Q4. It is important for me to know how the model makes its recommendation for my individual patient
- Q5. It is important for me to know how uncertain (in %) the model is about its recommendation

Questions related to the visual representation:

- Q6. The colour bar with the score is easy to interpret
- Q7. The colour bar with the score convinces me to accept or reject the model's recommendation
- Q8. The scatterplot with all patients is easy to interpret
- Q9. The scatterplot with all patients convinces me to accept or reject the model's recommendation
- Q10. The barplot with feature contribution is easy to interpret
- Q11. The barplot with feature contribution convinces me to accept or reject the model's recommendation

Questions related to the user's attitude towards the model:

- A. I am satisfied with the output information that CORONET provides
- B. CORONET helps me in making safe clinical decisions on patient management
- C. When my initial decision was the same as CORONET had recommended, I felt reassured
- D. I understand when and why CORONET may provide the wrong recommendation in some cases
- E. CORONET helps in cases where I am less confident in the decision on how to proceed
- F. Even when my initial course of action was different to what CORONET recommended, I still had full confidence in my original decision.
- G. I was surprised when CORONET recommended an action different to my own

Feedback from Healthcare Professionals

Below we quote received feedback regarding the usability of the tool from the Healthcare Professionals:

- *This could be a very useful tool to aid the clinician's decision. With every risk stratification score/tool - I'm used to using the MASCC score for febrile neutropenia, it helps your final decision. If my gut said admit, irrespective of any score, I would admit. Tools can be great to add confidence to a less obvious choice of admit/discharge.*
- *With all the patients I would have discharged home, I still would have asked my team to safety-net call for the next 10-14 days.*

- *I don't think this can be proposed in clinical practice*
- *decision to admit a cancer patient is complex and multifactorial - often dependent on social support, patient engagement and both cancer and treatment related complications*
- *Really good tool. Very easy to use. It would be great to get individual hospital trusts to consider adding it to hospital guidelines so doctors felt safer using it to base decisions off.*
- *Easy to use but differed a lot with my clinical assessment*
- *Having played with the app a little, it seems like the biggest impact on score comes from NEWS, so I think for many patients it won't add much, but can be a useful adjunct.*
- *An interesting concept but not one that I think would be used in its current format in clinical care-too formulaic and not individualized to patient risk factors*

Additionally, at the end of the experiment Healthcare Professionals were asked about aspects in which the tool could be improved in the future. Below we quote received feedback:

- *Inclusion in guidelines, clarify the legal implications*
- *CORONET is a standalone 'App' - to be useful (and used) it has to integrate with patient EPRs (Electronic Patient Record) so HCPs are not copying electronic information from EPR to CORONET - this not only takes time but prone to errors - This would be a major factor in my decision to recommend CORONET*
- *May be useful to separate high risk and low risk comorbidities. For example, some conditions such as COPD will cause more severe issues when combined with COVID than other conditions such as diabetes.*
- *It does not seem to take into account oxygen requirements independently of NEWS2; it is difficult to discharge someone who is needing 4L of oxygen!*
- *Take into consideration if they are active chemotherapy/immunotherapy. As even with a covid positive test, they still require iv antibiotics and monitoring. Higher risk of deterioration, especially lung cancers, if discharged home.*
- *The wording on the red/yellow diagram seems to be inconsistent re categories. 1st and 2nd are actions e.g. 'consider' admit/discharge, the 3rd is commenting on the disease severity. These are different categories, so the third should be more like 'consider level 2+ care' or another type of action*
- *The labeling of the red/yellow score is a bit hard to follow, suggest removing the bottom legend*

User interface design

The CORONET tool available at `https://coronet.manchester.ac.uk/` was developed following general principles of usability and universal design in order to meet level 2 (AA) of the Web Content Accessibility Guidelines (WCAG) 2.1 (`https://www.w3.org/TR/WCAG21/`). More details regarding the web accessibility are available at `https://www.manchester.ac.uk/web-accessibility/`.

Supplementary Tables

Table S.1: Tasks performed on computers at work by the healthcare professionals participating in the experiment.

	Advanced Nurse Practitioner or higher	Advanced Nurse Practitioner or higher	Consultant / attending physician	Consultant / attending physician	Consultant / attending physician	Consultant / attending physician	Consultant / attending physician	Doctor within first year of graduating (FY1) / intern	Doctor (within 2-4 years of graduating / specialty trainee (ST) / fellow	Doctor (within 2-4 years of graduating / specialty trainee (ST) / fellow	General practitioner / GPST / community doctor	General practitioner / GPST / community doctor	General practitioner / GPST / community doctor	Other (please enter text)	Registered Nurse	Specialist Nurse	Specialist registrar / ST 3+ / senior resident/senior fellow	Specialist registrar / ST 3+ / senior resident/senior fellow	Specialist registrar / ST 3+ / senior resident/senior fellow	Specialist registrar / ST 3+ / senior resident/senior fellow	Specialist registrar / ST 3+ / senior resident/senior fellow	Specialist registrar / ST 3+ / senior resident/senior fellow	Specialist registrar / ST 3+ / senior resident/senior fellow	
Emailing	x	x	x	x	x	x	x	x	x	x		x	x	x	x	x	x	x	x	x	x	x	x	96%
Accessing clinical guidelines	x	x	x	x	x	x	x	x	x	x		x	x	x		x	x	x	x	x	x	x	x	91%
Viewing test results	x	x	x	x	x	x	x	x	x	x	x	x	x	x		x	x	x	x	x	x	x	x	96%
Viewing drug charts	x	x	x	x	x	x	x	x		x			x	x		x		x		x	x	x	x	74%
Online clinical calculators		x	x	x			x	x	x	x	x	x	x	x	x		x	x	x	x	x	x	x	83%
Online scoring systems and/or algorithms	x	x	x	x			x	x		x		x	x				x	x	x	x		x	x	65%
Statistical analysis	x		x				x									x	x				x	x	x	35%

Table S.2: Responses related to ease of interpretation and convincing capacity of the tool's output. No significant difference between answers.

	median respons in Likert scale 1-7 [Q1,Q3]
Q6. The colour bar with the score is easy to interpret	6 [5,6]
Q7. The colour bar with the score convinces me to accept or reject the model's recommendation	4 [4,6]
Q8. The scatterplot with all patients is easy to interpret	5 [3.5,6]
Q9. The scatterplot with all patients convinces me to accept or reject the model's recommendation	5 [4,5.5]
Q10. The barplot with feature contribution is easy to interpret	5 [5,6]
Q11. The barplot with feature contribution convinces me to accept or reject the model's recommendation	5 [4,6]
	p>0.05 Friedman test

Table S.5: Responses to questions Q2-Q5 related to user expectation regarding the output of the recommendation tool. Answers to Q2 are significantly lower than to other questions. No significant difference between Q3,Q4,Q5.

	median response in Likert scale 1-7 [Q1,Q3]	
Q2. It is important for me to know the mathematics behind the model's recommendations	4 [2,5]	b

Table S.5 continued from previous page

	median response<br>in Likert scale 1-7 [Q1,Q3]	
Q3. It is important for me to know the features of my patient contribute to the model's recommendation	6 [5,6.5]	a
Q4. It is important for me to know how the model makes its recommendation for my individual patient	6 [5,6]	a
Q5. It is important for me to know how uncertain (in %) the model is about its recommendation	6 [5.5, 7]	a
	$p<0.001$<br>Friedman test	$p<0.001$<br>posthoc Kruskal-Wallis

Table S.3: Individual comparisons between reported ease of interpretation of the visuals and their convincing capacity. No significant differences; all p>0.05

			p (Kruskal–Wallis)
Q6. The diagram (colorbar) is easy to interpret	vs.	Q8. The diagram (dotplot) is easy to interpret	0.131
Q10. The diagram (barplot) is easy to interpret	vs.	Q6. The diagram (colour bar) is easy to interpret	0.716
Q10. The diagram (barplot) is easy to interpret	vs.	Q8. The diagram (dotplot) is easy to interpret	0.244
Q11. The diagram (barplot) convinces me to accept or reject the model's recommendation	vs.	Q7. The diagram (colour bar) convinces me to accept or reject the model's recommendation	0.387
Q11. The diagram (barplot) convinces me to accept or reject the model's recommendation	vs.	Q9. The diagram (dotplot) convinces me to accept or reject the model's recommendation	0.281
Q7. The diagram (colour bar) convinces me to accept or reject the model's recommendation	vs.	Q9. The diagram (dotplot) convinces me to accept or reject the model's recommendation	0.740

Table S.4: Spearman correlations between questions regarding ease of interpretation of the visuals or their convincing capacity (Q6-Q11) and questions regarding user competence in the task (D4) or expectations regarding the output (Q1-Q5). No significant correlations were found (Holm adjusted p>0.05).

Spearman correlation r	D4. Rate your knowledge on the management of patients with cancer who have developed COVID-19	Q1. I feel comfortable when using new technology	Q2. It is important for me to know the mathematics behind the model's recommendations	Q3. It is important for me to know the features of my patient contribute to the model's recommendation	Q4. It is important for me to know how the model makes its recommendation for my individual patient	Q5. It is important for me to know how uncertain (in %) the model is about its recommendation
Q6. The colour bar with the score is easy to interpret	0.026	-0.101	-0.199	-0.109	-0.003	-0.187
Q7. The colour bar with the score convinces me to accept or reject the model's recommendation	-0.214	0.062	-0.195	0.031	0	-0.011
Q8. The scatterplot with all patients is easy to interpret	-0.105	0.333	-0.122	-0.309	0.085	-0.107
Q9. The scatterplot with all patients convinces me to accept or reject the model's recommendation	-0.156	0.433	-0.054	-0.283	0.059	-0.167
Q10. The barplot with feature contribution is easy to interpret	-0.368	0.077	-0.288	-0.06	-0.164	-0.044
Q11. The barplot with feature contribution convinces me to accept or reject the model's recommendation	-0.255	0.388	-0.268	-0.124	-0.038	-0.081

Table S.6: Responses to questions A-G after deciding on patient cases when supported by recommendation only (CS) and after decisions supported by recommendation and its explanation (CS+Exp). No statistically significant change observed in pairwise comparison (Wilcoxon signed-rank test).

Index	Question	Median response in Likert scale 1-7 [Q1,Q3]		Pairwise comparison	
		CORONET Score (CS)	CORONET Score + Explanation (CS+Exp)	p	significance*
A	I am satisfied with the output information that CORONET provides	5 [4.5,6]	5 [5,6]	0.976	ns
B	CORONET helps me in making safe clinical decisions on patient management	5 [3.5,5]	5 [4,5]	0.425	ns
C	When my initial decision was the same as CORONET had recommended, I felt reassured	5 [4,6]	5 [4,6]	0.746	ns
D	I understand when and why CORONET may provide the wrong recommendation in some cases	5 [3,6]	5 [4,5]	0.275	ns
E	CORONET helps in cases where I am less confident in the decision on how to proceed	4 [3,5]	5 [4,5.5]	0.056	ns
F	Even when my initial course of action was different to what CORONET recommended, I still had full confidence in my original decision	5 [5,6]	5 [5,6]	0.890	ns
G	I was surprised when CORONET recommended an action different to my own	4 [3,5]	4 [3,5]	0.821	ns

Table S.7: Statistically significant correlations between changes in responses between CS and CS+Exp scenarios. Only one after adjustment for multiple comparison (using Sidak correction).

Change in:	Change in:	r (spearman)	p	p-adjusted (Sidak)	significance
Reassurance	Understanding wrong recommendation	0.762	0.000	0.003	**
Satisfaction with the output	Help in making safe decisions	0.632	0.001	0.135	ns
Reassurance	Help when HCP is less confident	0.549	0.007	0.553	ns
Satisfaction with the output	Reassurance	0.513	0.012	0.771	ns
Help in making safe decisions	Help when HCP is less confident	0.418	0.047	0.997	ns

Supplementary Figures

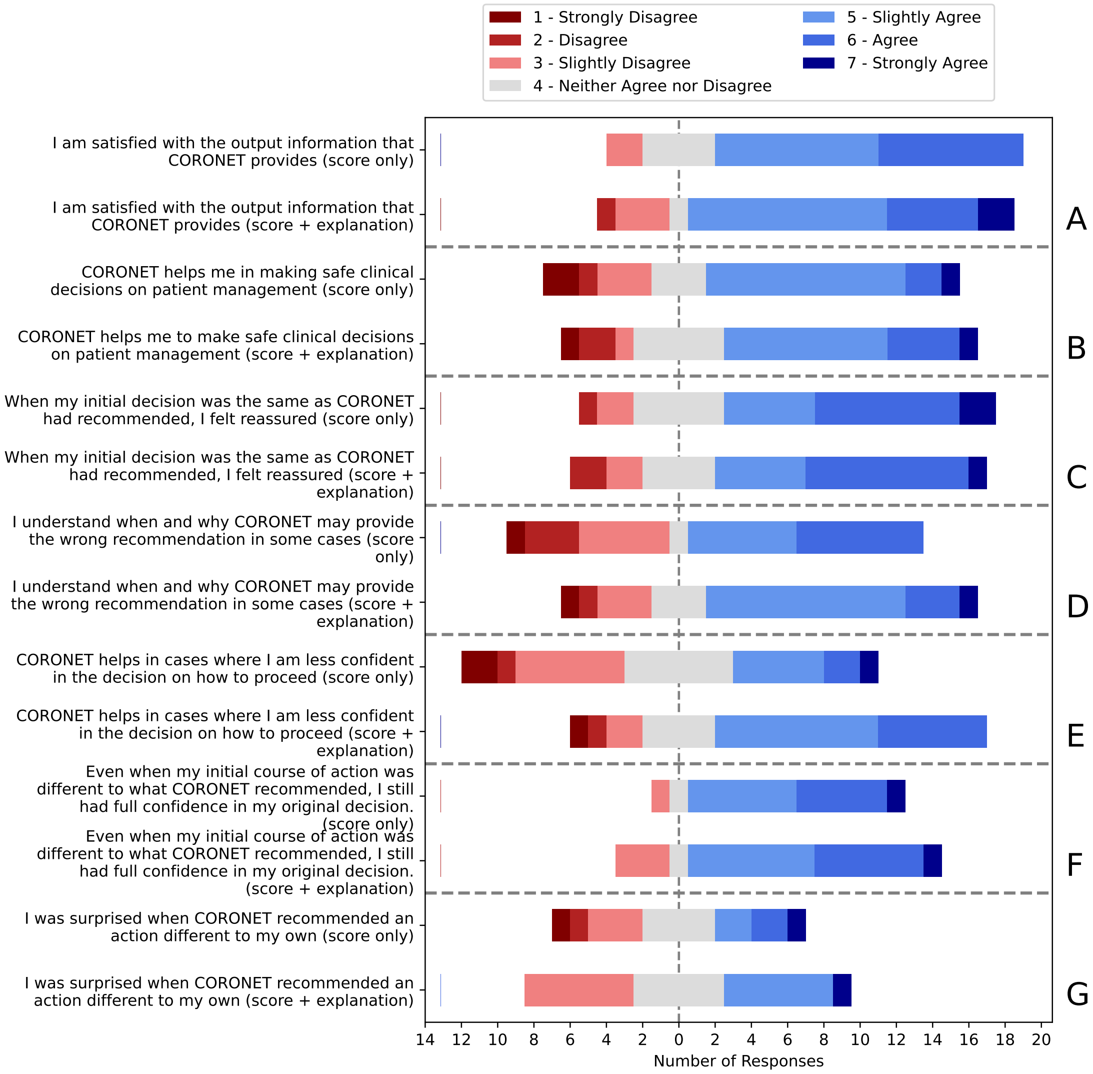

Figure S.1: Responses to questions A-G which were asked twice: after deciding on patients 1-5, where only the CORONET score (CS) was presented, and after deciding on the patients 6-10, when additional model's explanation was delivered (CS+Exp).

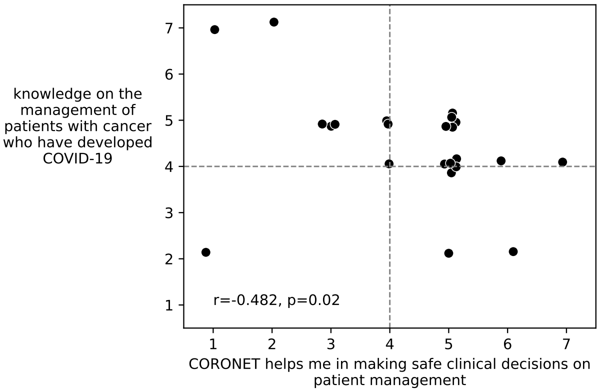

Figure S.2: Correlation indicating positive feedback to the model's recommendation, which is not supported by an explanation (CS). The lower the expertise, the more helpful the tool appeared to be, even when no explanation is provided. Answers on X axis are from CS scenario; points are scattered for visibility.

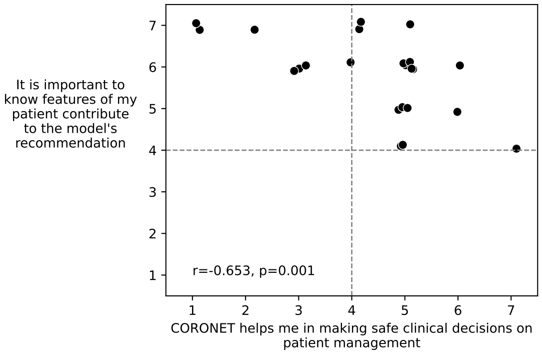

(a)

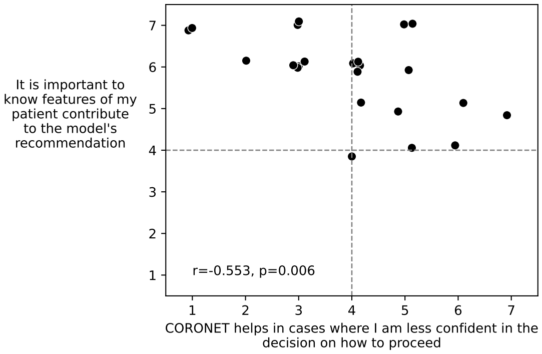

(b)

Figure S.3: Correlation indicating positive feedback to the model's recommendation, which is not supported by an explanation (CS). The higher the need for knowing the contributing features, the less helpful CS output is likely to be. Answers on X axis are from CS scenario; points are scattered for visibility.

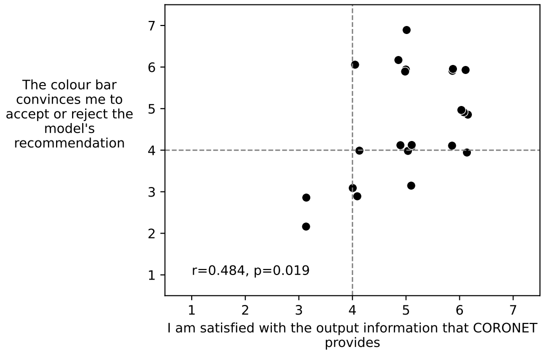

(a)

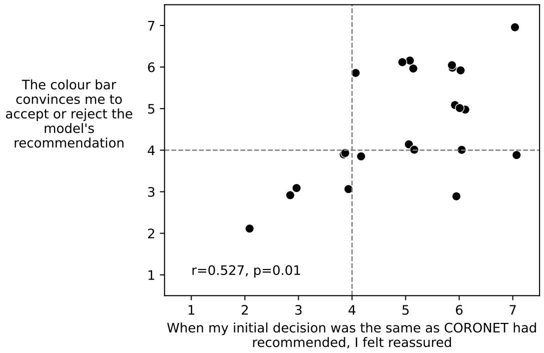

(b)

Figure S.4: Correlation indicating positive feedback to the model's recommendation, which is not supported by an explanation (CS). Satisfaction with the CS output, and reassurance when the tool recommended the same action as their own were correlated with being convinced by the colour bar. Answers on X axis are from CS scenario; points are scattered for visibility.

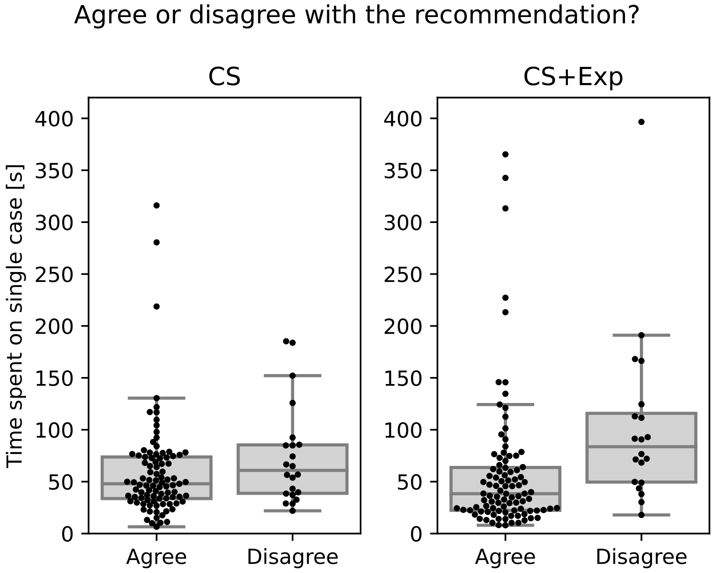

Figure S.5: Time spent on individual decisions for cases 1-5 (CS) and 6-10 (CS+Exp) stratified by the concordance between the user's final decision and the model's recommendation.

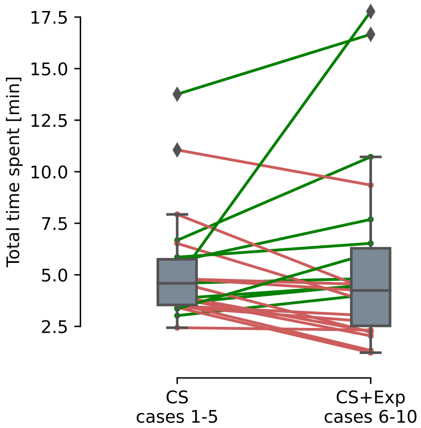

Figure S.6: Total time spent on making decisions about patients: cases 1-5 where only the score with colour bar were provided; cases 6-10 enhanced with two additional figures with explanation. Each line is an individual clinician. Green - time increased, red - decreased. No significant difference (p=0.846, Wilcoxon sign rank test).

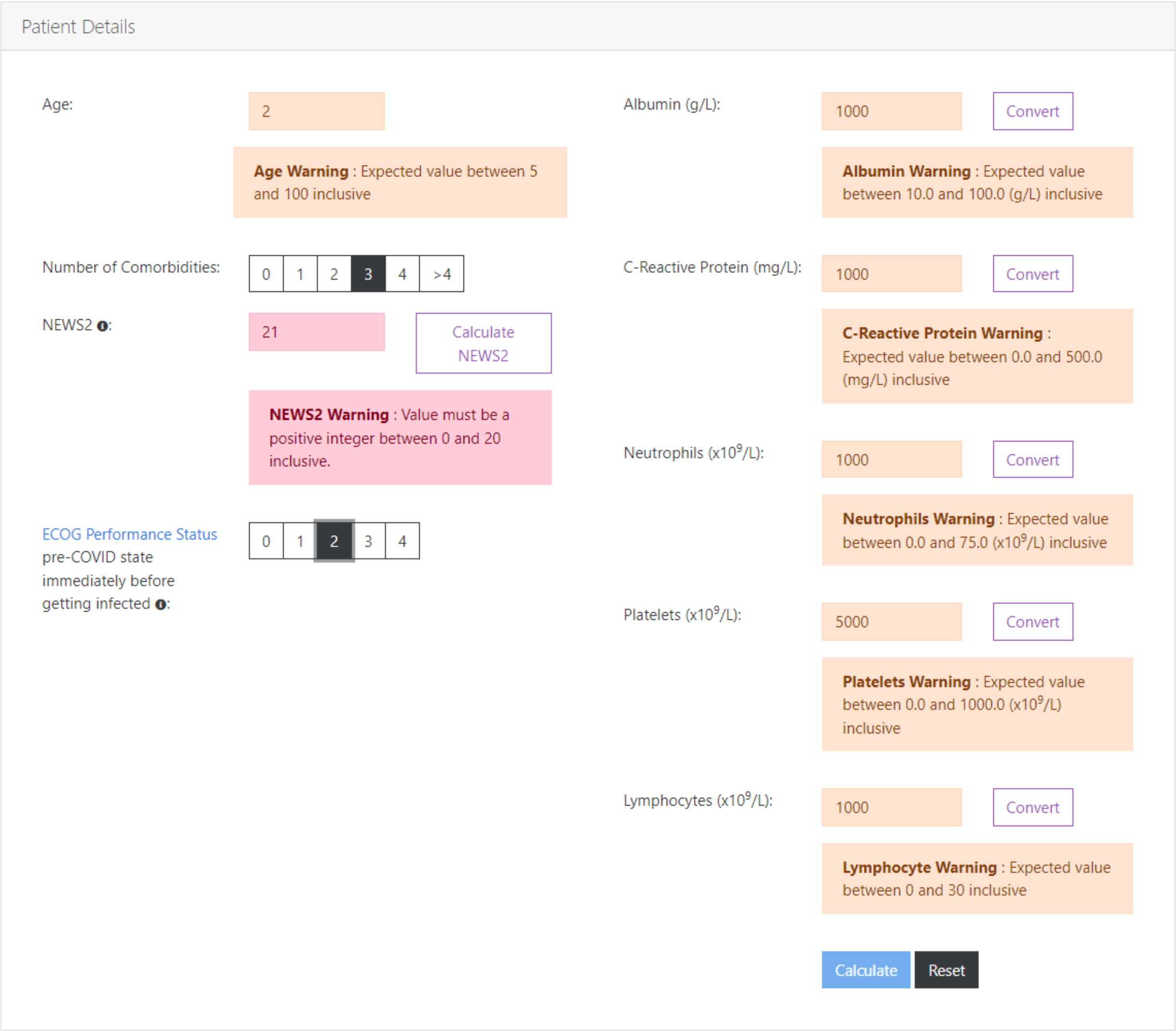

Figure S.7: Warnings displayed to the user when inputing the values of of expected range. user interface of the CORONET available at `https://coronet.manchester.ac.uk`.

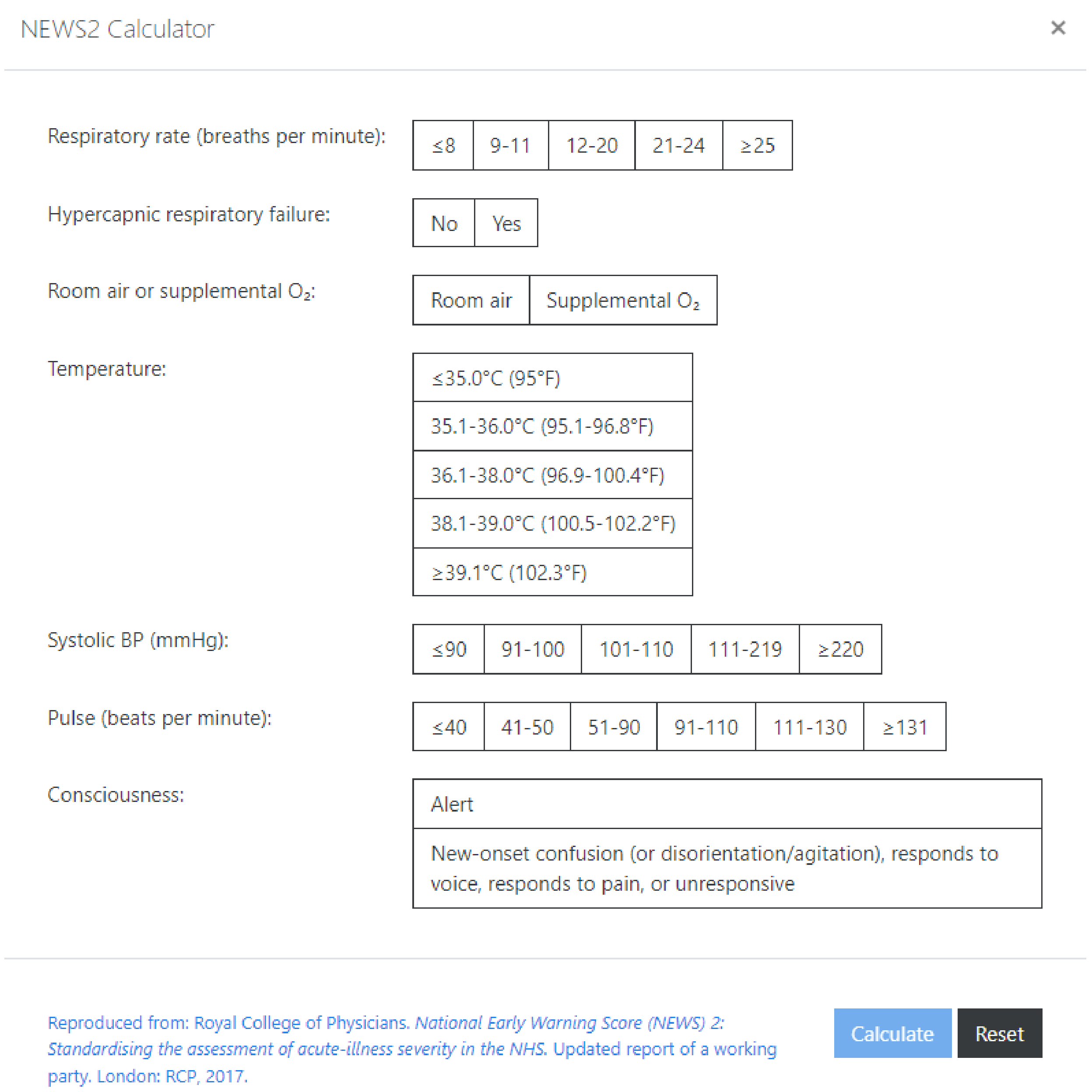

NEWS2 Calculator ×

Respiratory rate (breaths per minute):	≤8 | 9-11 | 12-20 | 21-24 | ≥25
Hypercapnic respiratory failure:	No | Yes
Room air or supplemental O_2:	Room air | Supplemental O_2
Temperature:	≤35.0°C (95°F) | 35.1-36.0°C (95.1-96.8°F) | 36.1-38.0°C (96.9-100.4°F) | 38.1-39.0°C (100.5-102.2°F) | ≥39.1°C (102.3°F)
Systolic BP (mmHg):	≤90 | 91-100 | 101-110 | 111-219 | ≥220
Pulse (beats per minute):	≤40 | 41-50 | 51-90 | 91-110 | 111-130 | ≥131
Consciousness:	Alert | New-onset confusion (or disorientation/agitation), responds to voice, responds to pain, or unresponsive

Reproduced from: Royal College of Physicians. *National Early Warning Score (NEWS) 2: Standardising the assessment of acute-illness severity in the NHS.* Updated report of a working party. London: RCP, 2017.

Calculate Reset

Figure S.8: A pop-window provided to the user when clicking on 'Calculate NEWS2' buttom. User interface of the CORONET available at `https://coronet.manchester.ac.uk`
